# Solution Equilibrium Studies on Salicylidene Aminoguanidine Schiff Base Metal Complexes: Impact of the Hybridization with L-Proline on Stability, Redox Activity and Cytotoxicity

**DOI:** 10.3390/molecules27072044

**Published:** 2022-03-22

**Authors:** Orsolya Dömötör, Nóra V. May, G. Tamás Gál, Gabriella Spengler, Aliona Dobrova, Vladimir B. Arion, Éva A. Enyedy

**Affiliations:** 1 Department of Inorganic and Analytical Chemistry, Interdisciplinary Excellence Centre, University of Szeged, Dóm tér 7, H-6720 Szeged, Hungary; domotor.o@chem.u-szeged.hu; 2 MTA-SZTE Lendület Functional Metal Complexes Research Group, University of Szeged, Dóm tér 7, H-6720 Szeged, Hungary; spengler.gabriella@med.u-szeged.hu; 3 Centre for Structural Science, Research Centre for Natural Sciences, Magyar Tudósok Körútja 2, H-1117 Budapest, Hungary; may.nora@ttk.hu (N.V.M.); gal.tamas@ttk.mta.hu (G.T.G.); 4 Department of Medical Microbiology, Albert Szent-Györgyi Health Center and Albert Szent-Györgyi Medical School, University of Szeged, Semmelweis u. 6, H-6725 Szeged, Hungary; 5 Institute of Inorganic Chemistry, University of Vienna, Währinger Strasse 42, A-1090 Vienna, Austria; aliona.luganschi@univie.ac.at (A.D.); vladimir.arion@univie.ac.at (V.B.A.)

**Keywords:** stability constants, aminoguanidine, EPR spectroscopy, anticancer, thiosemicarbazone

## Abstract

The proton dissociation processes of two tridentate salicylidene aminoguanidine Schiff bases (SISC, Pro-SISC-Me), the solution stability and electrochemical properties of their Cu(II), Fe(II) and Fe(III) complexes were characterized using pH-potentiometry, cyclic voltammetry and UV-visible, ^1^H NMR and electron paramagnetic resonance spectroscopic methods. The structure of the proline derivative (Pro-SISC-Me) was determined by X-ray crystallography. The conjugation of L-proline to the simplest salicylidene aminoguanidine Schiff base (SISC) increased the water solubility due to its zwitterionic structure in a wide pH range. The formation of mono complexes with both ligands was found in the case of Cu(II) and Fe(II), while bis complexes were also formed with Fe(III). In the complexes these tridentate ligands coordinate via the phenolato O, azomethine N and the amidine N, except the complex [Fe(III)L_2_]^+^ of Pro-SISC-Me in which the (O,N) donor atoms of the proline moiety are coordinated beside the phenolato O, confirmed by single crystal X-ray crystallographic analysis. This binding mode yielded a stronger Fe(III) preference for Pro-SISC-Me over Fe(II) in comparison to SISC. This finding is also reflected in the lower redox potential value of the iron-Pro-SISC-Me complexes. The ligands alone were not cytotoxic against human colon cancer cell lines, while complexation of SISC with Cu(II) resulted in moderate activity, unlike the case of its more hydrophilic counterpart.

## 1. Introduction

**Scheme 1:**
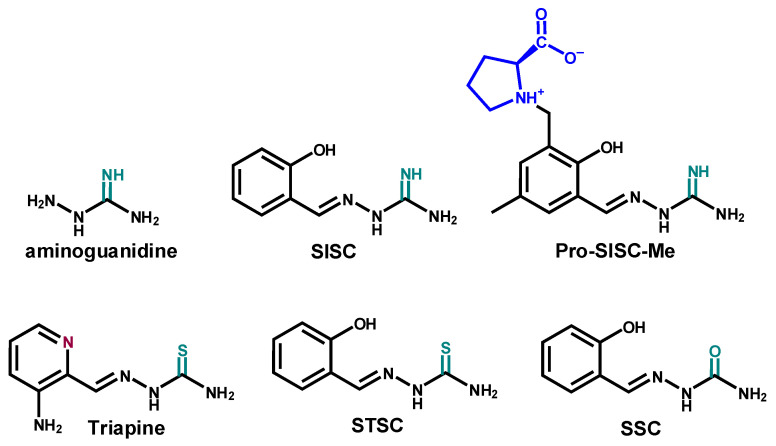
Chemical structures of aminoguanidine (also known as pimagedine), SISC, Pro-SISC-Me, the analogous salicylaldehyde (thio)semicarbazone STSC and SSC and Triapine in their neutral forms.

Aminoguanidine (also known as pimagedine, Scheme 1) is an α,β-dicarbonyl scavenging agent and was developed for the treatment of diabetic kidney disease, due to its ability to reduce the levels of advanced glycation end products which manages diabetic nephropathy [1]. The guanidine fragment appears in proteins as it is found in the side chain of arginine, and is generally positively charged at physiological pH; however, the guanidinium pK_a_ can be varied by the linked molecular moieties. Aminoguanidine can form Schiff bases with aldehydes or ketones, both in vivo (e.g., with pyridoxal phosphate [2]), and in vitro as reported in numerous papers [3,4,5,6,7]. Schiff bases (imines) and their transition metal complexes are widely investigated and considered as key classes of medicinal compounds due to their enormous potential for pharmacological activities such as anticancer, antibacterial and antioxidant effects [8,9]. Among the metal complexes of aminoguanidine-based Schiff bases, the Cu(II) complexes formed with salicylidene aminoguanidine or pyridoxilidene aminoguanidine are the most common [3,4,6,10,11], although we can find examples for V(V), Co(III) or Ni(II) complexes as well [5,6,10,12,13]. The Cu(II) complex of the (N,N,O) donor containing a Schiff base of aminoguanidine formed with salicylaldehyde (2-(2-hydroxybenzylidene)hydrazinecarboximidamide, SISC, Scheme 1) displayed significant cytotoxic activity on a colorectal cancer cell line (HCT1) and strong DNA-binding affinity [3]. The Cu(II) complex formed with the (N,N,N) donor containing a Schiff base of aminoguanidine and 2-acetylpyridine showed moderate and slight activity toward bacteria and toward molds, respectively [6].

Thiosemicarbazones (TSC) are also Schiff base ligands possessing numerous pharmacological applications, and the tridentate derivatives often coordinate via (N,N,S) or (O,N,S) donor sets [14,15,16]. Triapine (Scheme 1) is the best-known representative of TSCs, which has been studied in ca. 30 phase I and II clinical trials as an anticancer drug [15,17], and is ready to enter phase III clinical trials [18]. Its activity is linked to the inhibition of the ribonucleotide reductase enzyme based on its complex formation ability with iron ions [19]. In our previous work, the significant effect of the variation of the chalcogen atom in the TSC scaffold from sulfur to oxygen (semicarbazone) or selenium (selenosemicarbazone) on the pK_a_ values and the Fe(II)-, Fe(III)- and Cu(II)-binding ability, in addition to the cytotoxicity, was reported [20]. It was found that the iron preference has a strong impact on the biological activity due to the suggested mechanism of action of terminally non-substituted TSCs such as Triapine (Scheme 1). e.g., the exchange of the thioamide sulfur to oxygen in Triapine resulted in a loss of cytotoxicity, most probably due to the inability of oxo-Triapine to bind iron ions efficiently in both oxidation states (+2 and +3). On the other hand, the Fe(III) preference of the salicylaldehyde TSC (STSC, Scheme 1) possessing (O,N,S) chelating moiety is also stronger over Fe(II) in comparison with the α-*N*-pyridyl TSCs (e.g., Triapine), which seems to be an unfavorable property in terms of the cytotoxic activity [21,22]. The salicylaldehyde semicarbazone (or salicylidene aminoguanidine, SSC, Scheme 1) with (O,N,O) donor set is also a non-cytotoxic compound [23]. Meanwhile, the Cu(II) complexes, both STSC and SSC, are reported to have moderate cytotoxicity [24,25,26].

In the present comparative study, SISC (Scheme 1), the aminoguanidine congener of STSC, was applied to investigate the effect of the =NH/=S exchange on the solution and biological properties. Additionally, its enantiomerically pure proline hybrid (R-1-(3-)((2-carbamimidoylhydrazono)methyl)-2-hydroxy-5-methylbenzyl)pyrrolidine-2-carboxylic acid, Pro-SISC-Me, Scheme 1) with increased water solubility was also synthesized and characterized. Therefore, the proton dissociation processes and lipophilicity of SISC and Pro-SISC-Me, the solution stability, stoichiometry and electrochemical properties of their Cu(II), Fe(II) and Fe(III) complexes, were studied and compared to the analogous TSCs. The relationship between these data and the cytotoxicity of the compounds in Colo-205 (human colon cancer), Colo-320 (doxorubicin-resistant human colon cancer) and normal human embryonal lung fibroblast (MRC-5) cells was also examined.

## 2. Results and Discussion

### 2.1. Synthesis and Characetriazion of Pro-SISC-Me

**Figure 1:**
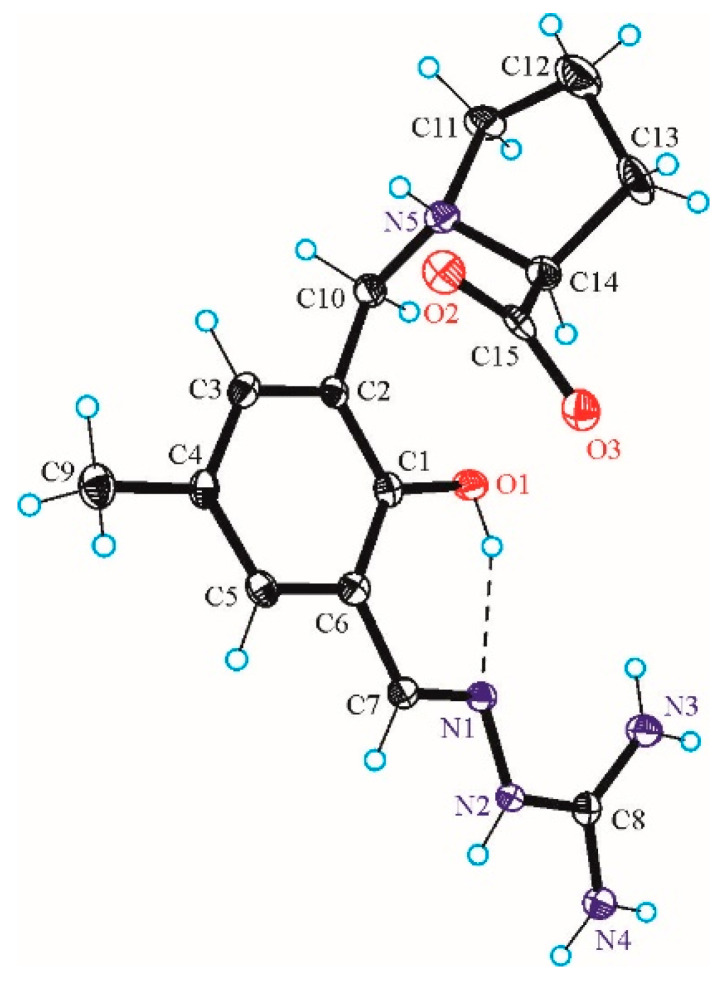
ORTEP view of Pro-SISC-Me·HBr (**1**) with thermal ellipsoids at 50% probability level. The counter ions and solvent molecules are omitted for clarity. Selected bond distances (Å) and torsion angles (deg): C1–O1 1.359(5), N1–N2 1.371(5), N2–C8 1.338(6), C8–N3 1.325(6), C8–N4 1.335(6); C1–C2–C10–N5 –101.1(4); C10–N5–C14–C15 –90.0(4).

The chiral compound Pro-SISC-Me·HCl·2H_2_O was prepared by condensation reaction of 2-hydroxy-3-methyl-(*S*)-pyrrolidine-2-carboxylate-5-methylbenzaldehyde with in situ prepared aminoguanidinium chloride in boiling methanol/water in 61% yield. The formation of the Schiff base was confirmed by elemental analysis, as well as by single crystal X-ray diffraction (SC-XRD) analysis of the aminoguanisonium bromide salt (Pro-SISC-Me·HBr (**1**)) obtained similarly to the chloride salt (Pro-SISC-Me·HCl·2H_2_O). The result of X-ray diffraction study of **1** is shown in Figure 1 with selected metric parameters quoted in the legend. Even though the series of transition metal complexes formed with TSCs derived from the same aldehyde or 2-formylpyridine coupled via a methylene group to L-proline moiety is well documented in the literature [27,28,29,30,31,32], there is only one structural characterization of a metal-free TSC [28].

As in 2-hydroxy-3-methyl-(*S*)-pyrrolidine-2-carboxylate-5-methylbenzaldehyde 4-*N*-pyrrolidine-3-thiosemicarbazone [28], the L-proline moiety in Pro-SISC-Me·HBr adopts a zwitterionic form, which similarly makes the atom N5, in addition to C14, chiral. Intramolecular hydrogen bond O1–H···N1 [O1···N1 2.670(5) Å and O1–H···N1 145.4°] is also found, which is typical for this kind of Schiff base [5,10].

### 2.2. Solution Chemistry of SISC and Pro-SISC-Me

The studied compounds (Scheme 1) have relatively high thermodynamic solubility at pH 7.4 (S_7.4_~2 mM (SISC), S_7.4_~10 mM (Pro-SISC-Me), T = 25 °C, 10 mM HEPES); however, their metal complexes possess worse solubility in water. Therefore, most of the solution equilibrium studies were performed in 30% (*v*/*v*) DMSO/H_2_O solvent mixture in this work. Meanwhile, the proton dissociation processes of the ligands were also monitored in aqueous solutions. The proton dissociation constants (pK_a_) were determined by pH-potentiometric, UV-visible (UV-vis) and ^1^H NMR spectroscopic titrations and are collected in Table 1. Two pK_a_ values could be determined for SISC by pH-potentiometry, while for the proline hybrid an additional proton dissociation process (characterized by pK_1_) was detected in the studied pH range (2–12). The fundamental difference between the TSCs (-C=N-NH-C(=S)NH_2_) and the SCs (-C=N-NH-C(=O)NH_2_) is that the iminosemicarbazone moiety (-C=N-NH-C(=NH)NH_2_) is a basic group resulting in a positive charge when it becomes protonated.

**Figure 2:**
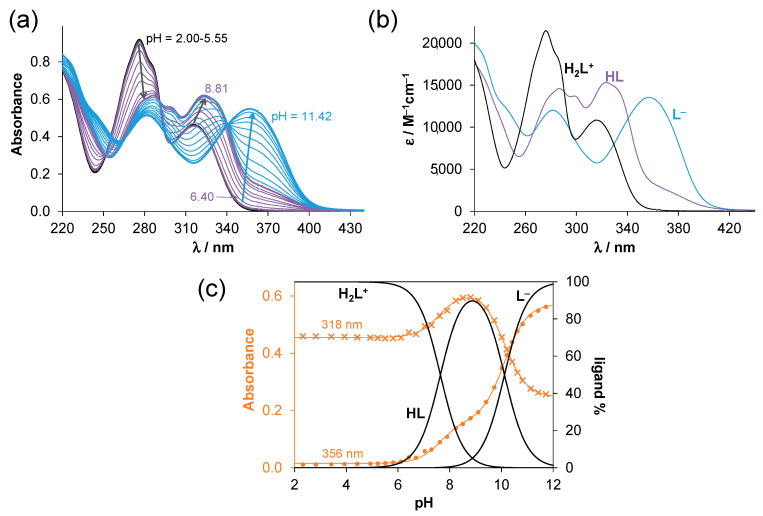
(**a**) UV-vis absorption spectra of SISC recorded at various pH values in water; and (**b**) individual molar absorbance spectra of the ligand species in the different protonation states; (**c**) Concentration distribution curves with absorbance values measured at 318 nm (×) and 356 nm (●) with the fitted curves. (*c*_ligand_ = 42 µM; *T* = 25 °C; *I* = 0.10 M (KCl); *ℓ* = 1 cm).

UV-vis and ^1^H NMR spectra were recorded at different pH values for both ligands. The UV-vis spectra of SISC (Figure 2 and Figure S1) and Pro-SISC-Me (Figure S2) reveal very similar, although not identical, spectral changes upon increasing the pH, which suggests that the same functional groups of the ligands deprotonate in each step. Taking into account the pH dependence of the absorbance values (Figure 2c, Figure S1c and Figure S2c), two deprotonation steps can be observed. The molar absorbance spectra of the ligand species in the different protonation states (H_2_L^+^, HL, L^−^) can be seen in Figure 2b, Figure S1b and Figure S2b. These individual spectra show that both deprotonation steps are accompanied by a significant red shift, especially in the case of the HL → L^−^ process. The increased λ_max_ has often originated from a more extended conjugated π-electron system, which is typically observed for the deprotonation of phenolic OH groups [22,24]. Notably, the deprotonation of the nonchromophoric proline moieties in Pro-SISC-Me (COOH, N_Pro_H^+^) is not expected to result in significant spectral changes. Thus, pK_1_ of Pro-SISC-Me (which could be determined by pH-potentiometry) belongs to the carboxylic acid group. 

**Figure 3:**
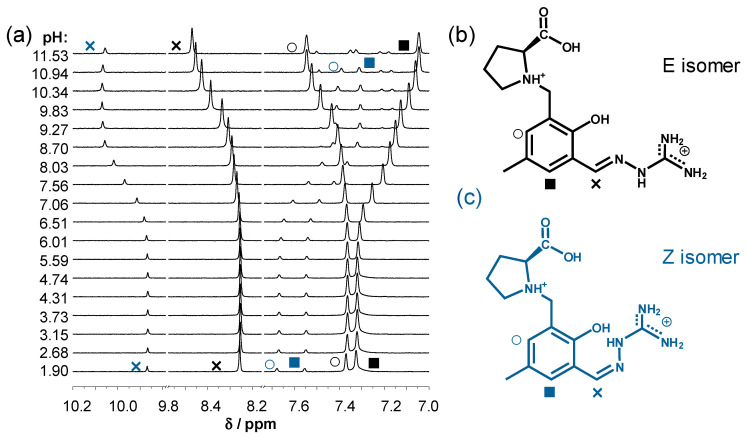
(**a**) ^1^H NMR spectra of Pro-SISC-Me in the low field region recorded in aqueous medium at the indicated pH values, and structure for the H_3_L^2+^ forms of the (**b**) E and the (**c**) Z isomers with the notation of the symbols of the CH protons of the salicylaldehyde aminoguanidine moiety assigned to the peaks in the NMR spectra in (**a**). (*c*_ligand_ = 1.0 mM; 10% (*v*/*v*) D_2_O/H_2_O; pH = 2.2–12.2; *T* = 25 °C; *I* = 0.10 M (KCl)).

^1^H NMR spectra recorded for SISC in 30% (*v*/*v*) d_6_-DMSO/H_2_O (Figure S3) clearly show two deprotonation steps and all the CH protons are sensitive to the changes of the pH. Similarly, the changes in the chemical shifts of the peaks of the aromatic CH and the CH=N protons indicate two steps in the ^1^H NMR spectra of Pro-SISC-Me recorded in water (Figure 3a), thus two pK_a_ values were calculated (pK_2_, pK_3_ in Table 1). However, this is only true for the major species identified as the E isomer (Figure 3b), while for the other isomer (Z, Figure 3c) only one pK_a_ (7.55 ± 0.02) could be determined. The E and Z geometric isomers are the consequence of the C=N double bond. A possible hydrogen bond between the deprotonated phenolato oxygen and the hydrazonic NH might result in an increased pK_a_ of the aminoguanidinium moiety (>11.5) and decreased pK_a_ of the OH. Based on the integrals of the CH=N peaks, the fraction of the E isomer is 86% at pH < 6.5, and it is decreased to 82% reaching pH 11.5; however, the appearance of further minor peaks at pH > 9.8 indicates the presence of other species in low fraction. The same picture is seen when the changes of the CH_3_ peaks are considered (Figure S4), and only the peak of the CH-COOH proton was sensitive to the first deprotonation process at pH < 3, which confirms that pK_1_ of Pro-SISC-Me applies to COOH. In the presence of 30% (*v*/*v*) DMSO only one type of isomer is present (most probably E), or there is a fast exchange process between the isomers (in the case of SISC only one type of isomer was observed in both media).

The pK_a_ of H_2_L^+^ is only slightly affected by the presence of the DMSO in the case of SISC, while for Pro-SISC a minor decrease is seen. The pK_a_ of HL becomes higher in the case of both ligands, indicating that the HL forms are better solvated in the DMSO/H_2_O mixture than in pure water. It suggests that the HL form of SISC and Pro-SISC-Me is neutral, as they are shown in Scheme 1. Therefore, the deprotonation of HL is more likely to be assigned to the OH group; however, the proton dissociation processes of H_2_L^+^ and HL are partly overlapped. This is the reason why it is difficult to distinguish between the deprotonation of the phenolic OH and the aminoguanidinium moiety. 

Based on the determined pK_a_ values, the proline residue in Pro-SISC-Me is zwitter ionic (COO^−^, N_Pro_H^+^) in a wide pH range (pH = 4.5–11.5, see the concentration distribution curves in Figure S2c); and its presence in the molecule results in lower pK_a_ values in comparison to those of SISC, especially in the case of pK_a_ of HL (~0.5 log unit), which can be the result of the electron withdrawing effect of the N_proline_H^+^ moiety and a possible intermolecular hydrogen bond between the deprotonated phenolato oxygen and the N_proline_H^+^ group.

Comparing the pK_a_ values of SISC to those of the analogous salicylaldehyde TSC (STSC, pK_1_ = 8.89; pK_2_ = 12.59 [22]) and SC (SSC, pK_1_ = 9.32; pK_2_ > 12.8 [23]) (Scheme 1) in 30% (*v*/*v*) DMSO/H_2_O, it can be concluded that the exchange of the sulfur in the thioamide moiety of the TSC or the carbonyl oxygen in the SC to the NH group in the aminoguanidine significantly affects the pK_a_ values, thus the actual chemical forms at a given pH. As a consequence, 100% and 97% of SSC and STSC, respectively, are present in their neutral form at pH 7.4; while at this pH, for SISC 64% H_2_L^+^ and 36% HL and for Pro-SISC-Me 33% H_2_L^+^ and 67% HL are found. It is noteworthy that HL is neutral but zwitter ionic in the case of Pro-SISC-Me. The different distribution of the species and charges are expected to result in different lipophilicity of the compounds. Distribution coefficients (logD_7.4_) were determined for the aminoguanidine ligands by *n*-octanol/water partitioning at pH 7.4 (Table 1, Figure S5). The obtained data for SISC (+0.83) and Pro-SISC-Me (−0.88) are lower than those of STSC (+1.59 [24]) and SSC (+0.94 [23]) due to the presence of the partly positively charged aminoguanidinium moiety and the zwitter ionic amino acid residue in Pro-SISC-Me. Based on these data, the incorporation of the proline residue resulted in a much stronger hydrophilic character as it was also observed e.g., for the proline hybrids of STSC [27], 2-formylpyridine TSC [30] or 8-hydroxyquinolines [33].

It was also found that both compounds possess intrinsic fluorescence at pH 7.4 (see λ_EX_ and λ_EM_ values in Table 1, and Figure S6) similar to the analogous TSC or SC compounds [22,23,24], and the Pro derivatization resulted in stronger emission intensity. Interestingly, the fluorescence excitation spectrum of Pro-SISC-Me resembles the absorption spectrum of the L^−^ form and also SISC can be excited at wavelengths (350–400 nm) which are typical for the phenolate form. 

### 2.3. Complex Formation of SISC and Pro-SISC-Me with Cu(II) Ions

Interaction between Pro-SISC-Me and Cu(II) ions could be studied by pH-potentiometry in water due to the adequate solubility of the species (Figure S7b), and the calculated overall stability constants are collected in Table 2. Based on the titration data, formation of only mono-ligand complexes ([CuL]^+^, [CuLH_−1_], [CuLH_−2_]^−^) was observed even at higher metal-to-ligand ratios. Stability constants for the complexes formed with the more lipophilic SISC were determined in the presence of 30% (*v*/*v*) DMSO/H_2_O (Figure S7a), and for the Cu(II)—Pro-SISC-Me system was studied as well in the same solvent system for comparison (Table 2). Notably, [CuL]^+^ is formed by the displacement of two protons from the ligand, therefore, coordination of both the phenolato and the aminoguanidine moieties is probable in this species. In the case of SISC, the stability constant could not be obtained for the [CuLH_−2_]^−^ species as in its formation pH range (>8) precipitation appeared in the solution. The thiosemicarbazone analogues (STSC and Pro-STSC-Me) also form exclusively mono-ligand complexes ([CuLH]^+^, [CuL], [CuLH_−1_]^−^) [22,27], however, protonated complexes could not be found for SISC and Pro-SISC-Me. 

**Figure 4:**
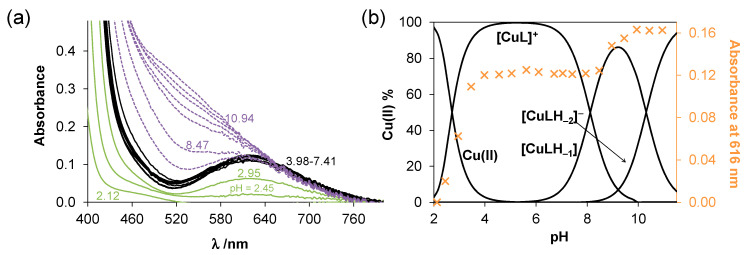
(**a**) UV-vis absorption spectra recorded for the Cu(II)—Pro-SISC-Me (1:1) system at various pH values; (**b**) Concentration distribution curves calculated under the same conditions with absorbance values measured at 616 nm (×). (*c*_ligand_ = *c*_Cu(II)_ = 1.0 mM; 30% (*v*/*v*) DMSO/H_2_O; *T* = 25 °C; *I* = 0.10 M (KCl); *ℓ* = 2 cm).

Complex formation was also monitored by UV-vis spectrophotometric and EPR titrations to confirm the speciation model determined by pH-potentiometry and to reveal the coordination modes. The recorded UV-vis spectra (Figure 4a, Figure S8a and Figure S9a) show characteristic changes upon increasing the pH. Namely, the development of a d-d band with λ_max_ 616 nm was observed in both systems parallel to the formation of the [CuL]^+^ complex, indicating similar coordination modes (Figure 4b and Figure S8b), and the spectra remained intact in a broad pH range (4.5–7.5). Further changes were seen for the Cu(II)—Pro-SISC-Me system in the basic pH range in the wavelength range of both the ligand and d-d bands (Figure 4a and Figure S9a), while in the case of SISC the precipitate formed even at the applied lower concentrations (ca. 0.5 mM, Figure S8a). This finding suggests further changes in the coordination modes, which resemble to the spectral changes reported for the formation of the mixed hydroxido [CuL(OH)]^−^ complexes of Pro-STSC-Me [27]. Thus, complexes formed in the basic pH range are most likely mixed hydroxido species ([CuLH_−1_] = [CuL(OH)] and [CuLH_−2_]^−^ = [CuL(OH)_2_]^−^). 

**Figure 5:**
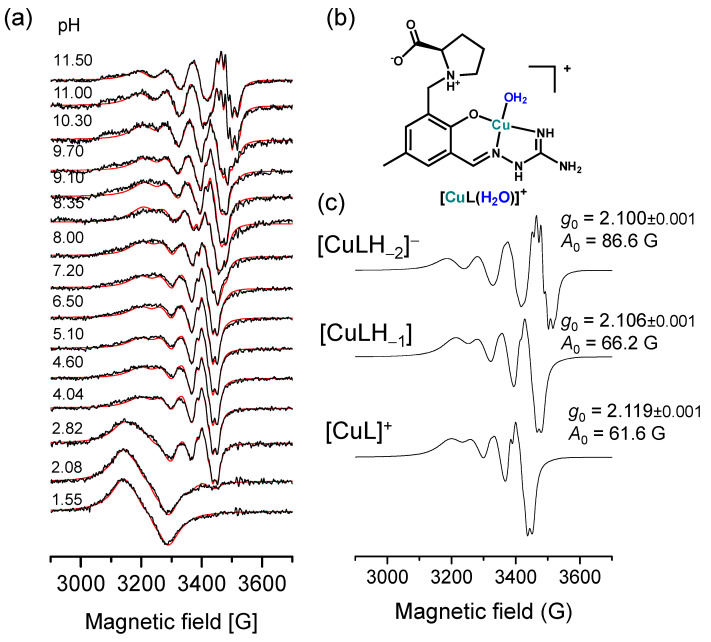
(**a**) Experimental (black) and simulated (red) isotropic EPR spectra recorded for the Cu(II)—Pro-SISC-Me (1:1) system in water at room temperature at various pH values; (**b**) Suggested structure of the [CuL(H_2_O)]+ complex (the possible hydrogen bonding between the proline nitrogen and the phenolato oxygen is not marked in the figure as well as the coordinated solvent molecules in the axial positions); (**c**) The calculated component EPR spectra and the g_0_ and A_0_ values of the species. (*c*_ligand_ = *c*_Cu(II)_ = 1.0 mM; *I* = 0.10 M (KCl)).

EPR spectra were recorded for the Cu(II)—Pro-SISC-Me species at room temperature in aqueous solution (Figure 5a) and two-dimensional simulation of the solution EPR spectra resulted in the individual isotropic EPR spectra (Figure 5c) and parameters (Table S1). Deconvolution of the EPR spectra clearly shows the formation of three different types of complexes: [CuL]^+^, [CuLH_−1_] and [CuLH_−2_]^−^, characterized by decreasing g_0_ and increasing A_0_ parameters. The nitrogen hyperfine splitting, caused by the equatorial coordination of two nitrogen donor atoms, is well-resolved in all component spectra. The two nitrogens were not equivalent, most probably the middle nitrogen (N^1^) has a higher coupling. No dinuclear or bis-ligand complexes were found under these conditions. The EPR parameters also show the regular elongated octahedral geometry around the Cu(II) ion. Based on these data, we concluded that in [CuL]^+^, coordination of the ligand via phenolato oxygen, azomethine N and one of the guanidine nitrogens is the most likely (Figure 5b), and the other two species are mixed hydroxido species. It should be noted that hydrogen bonding is possible between the proline nitrogen and the phenolato oxygen as it was found by X-ray crystallography for the Cu(II)-Pro-STSC-Me complex [27]. 

EPR spectra for the Cu(II)—SISC species were recorded at 77 K (Figure S10a) since the solubility did not allow the measurements at room temperature which requires higher concentrations. All component EPR spectra could be fitted by taking into account rhombic g- and A-tensors (Figure S10b, Table S2) and the superhyperfine splitting of two nitrogens coordinating in the equatorial plane. The [CuL]^+^ complex shows slight rhombic distortion, probably due to the attached chelate rings. The deprotonation of this complex does not affect the coordination sphere and it causes only slight changes in the EPR spectra, similarly as it was seen for the Pro-SISC-Me species. Precipitation occurred at pH > 8 and the spectral intensity decreased, although at pH ca. 12 the solution became clear again owing to the formation of a mixed hydroxido complex ([CuL(OH)_2_]^−^). The superhyperfine lines of this spectra were very well resolved, which made it possible to determine the nitrogen couplings with high accuracy (Table S2). The average of the g parameters of the SISC complexes (g_0,calc_. in Table S2) were close to those of the directly determined values of the Pro-SISC-Me species, suggesting similar coordination modes in the corresponding complexes. Notably, a dimeric complex of SISC with perchlorate counter ions [(CuLH)_2_](ClO_4_)_2_ was prepared and crystallized out from a methanolic solution by Mondal et al. [3]. The X-ray crystallographic analysis of this complex revealed that the ligands coordinated tridentately via the phenolato O, azomethine N and the amidine N, and the fourth coordination position was occupied by a bridging phenoxide O from the ligand bound to the second Cu(II) of the binuclear unit [3]. However, there was no indication for the presence of such dimeric species in the 30% (*v*/*v*) DMSO/H_2_O solvent mixture based on the EPR spectroscopic measurements. 

Based on the stability constants, it can be concluded that [CuL]^+^ complexes predominate at pH 7.4 (SISC: 91%, Pro-SISC: 100%). The logD_7.4_ values were determined for the Cu(II) complexes, and −0.49 ± 0.03 and −0.87 ± 0.03 were obtained for the complex of SISC and Pro-SISC-Me, respectively, revealing their fairly hydrophilic character due to their positive charge. The formation of precipitate in the Cu(II)—SISC system was due to the neutral [CuL(OH)]. For the comparison of the complex stability at pH 7.4, pCu values (the negative decadic logarithm of the equilibrium concentration of the free metal ion, Table 2) were calculated. A higher pCu indicated stronger Cu(II) affinity of the ligand. These values show the negligible dissociation of the complexes under this condition; therefore, they are considered to be a highly stable species and their stability is comparable to that of the tridentate thiosemicarbazone complexes. The Cu(II) binding ability of the proline derivative was somewhat higher in comparison to the reference compound SISC. A similar trend was reported for STSC and Pro-STSC-Me [22,27]. 

**Figure 6:**
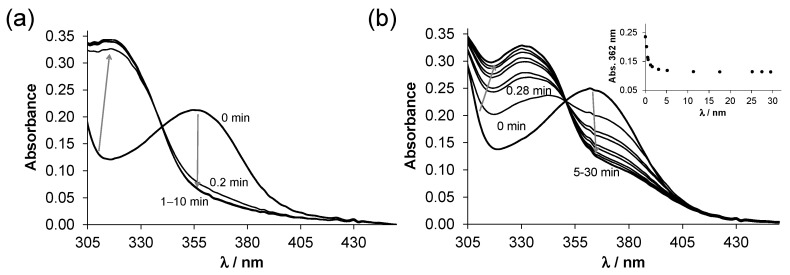
(**a**) Time-dependent changes of the UV–vis absorption spectra of (**a**) the Cu(II)—SISC (1:1); and (**b**) the Cu(II)—Pro-SISC-Me (1:1) systems in the presence of 10 equiv. GSH at pH 7.4 in 30% (*v*/*v*) DMSO/H_2_O under argon. Inserted figures in (**b**) show absorbance changes at 362 nm (●). (*c*_Cu(II)_ = *c*_ligand_ = 25 μM, *c*_GSH_ = 250 μM; pH = 7.4 (20 mM HEPES); *T* = 25 °C; *I* = 0.10 M (KCl); *ℓ* = 1 cm).

The redox properties of the Cu(II) complexes were investigated as a first step by cyclic voltammetry, and representative voltammograms are shown in Figure S11. Unfortunately, irreversible redox reactions were observed in the pH range of the complex formation, thus, the comparison of these data is not adequate, and prediction of their ability to be reduced by physiological reductants is not possible. Therefore, the direct redox reaction of these Cu(II) complexes with glutathione and ascorbic acid was studied at pH 7.4 in 30% (*v*/*v*) DMSO/H_2_O solvent mixture by UV-vis spectrophotometry using a tandem cuvette under anaerobic conditions. The mild reducing agent, ascorbate was not able to reduce the complexes. On the other hand, GSH resulted in significant spectral changes in the wavelength range 310–430 nm (Figure 6). Namely, the decrease of the absorbance at ca. 360 nm was observed, and absorbance was increased at the λ_max_ of the free ligand (316 nm (SISC), 330 nm (Pro-SISC-Me)). It indicates the dissociation of the ligand from the complex upon the reduction, while the forming Cu(I) most probably forms a complex with the GSH that is present in a high excess in the solution. Similar behavior was also reported for the Cu(II) complexes of STSC [24], however, the reaction rate differs. The redox equilibrium was reached within 1 min (SISC), 5 min (Pro-SISC-Me) and 13 min (STSC) under the applied condition (25 μM complex, 250 μM GSH). Therefore, similar to the Cu(II)-STSC complex [24], the complexes of SISC and Pro-SISC-Me are considered as redox active species under physiological relevant conditions, which may contribute to their cytotoxic activity. 

### 2.4. Complex Formation of SISC and Pro-SISC-Me with Fe(III) and Fe(II) Ions

As the iron binding ability of the (thio)semicarbazone-related compounds and the difference between the stability of the complexes formed Fe(II) and Fe(III) ions are assumed to have an impact on the biological activity, the stability constants for the complexes formed with SISC and Pro-SISC-Me were determined by the pH-potentiometric titrations in 30% (*v*/*v*) DMSO/H_2_O (Table 3). 

Complexation with Fe(II) was studied under strictly anaerobic conditions. The obtained titrations’ curves (see Figure S12a for the Fe(II)—SISC system) show that the complex formation starts at relatively high pH values (>5.5) with both ligands, and, in the case of SISC precipitate, occurred at pH > 9 at 1:1 and at pH > 10 at 1:2 metal-to-ligand ratios. Evaluation of the titration data revealed that only mono complexes were formed in different protonation states (Figure S12b) as was also found with the Cu(II) ions (vide supra), although the Fe(II) complexes have a much lower stability. Aside from the better solubility of the Pro-SISC-Me complexes, the two ligands behaved similarly and the difference between their Fe(II)-binding efficacy was minor. 

**Figure 7:**
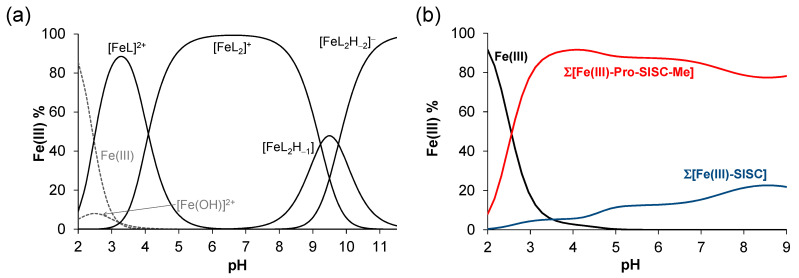
(**a**) Concentration distribution curves of the Fe(III)—Pro-SISC-Me (1:2) system in 30% (*v*/*v*) DMSO/H_2_O. (*c*_ligand_ = 1 mM; *T* = 25 °C; *I* = 0.10 M (KCl)); (**b**) Predominance curves calculated for the hypothetic Fe(III)—SISC—Pro-SISC-Me (1:2:2) system on the basis of the overall stability constants to reveal the ligand preference of Fe(III). (*c*_Fe(III)_ = 0.5 mM; *T* = 25 °C; *I* = 0.10 M (KCl)).

**Figure 8:**
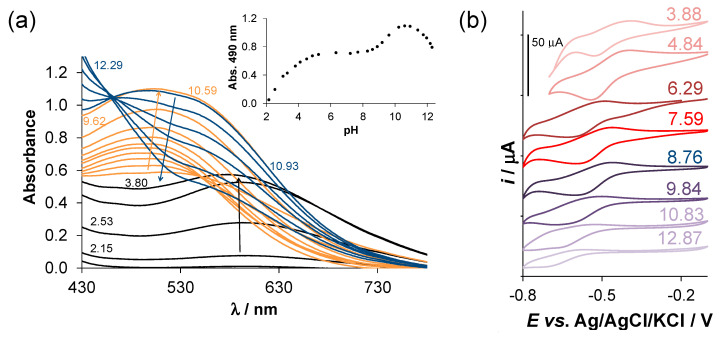
(**a**) UV-vis spectra recorded for the Fe(III)—Pro-SISC-Me (1:2) system in 30% (*v*/*v*) DMSO/H_2_O at various pH values (indicated in the figure). (*c*_ligand_ = 1 mM; *T* = 25 °C; *I* = 0.10 M (KCl); *ℓ* = 1 cm); (**b**) Cyclic voltammograms for the iron complexes of Pro-SISC-Me at 1:2 metal-to-ligand ratio at various pH values in 30% (*v*/*v*) DMSO/H_2_O. (*c*_ligand_ = 1 mM; *T* = 25 °C; *I* = 0.10 M (TBAN); scan rate: 20 mV/s).

Unlike Fe(II), the Fe(III) ions formed bis-ligand complexes as well under the conditions studied. The overall stability constants determined for the [Fe(III)L]^2+^ and [Fe(III)L_2_]^+^ complexes (Table 3) are similar for the two ligands; however, formation of [Fe(III)L_2_H_−1_] and [Fe(III)L_2_H_−2_]^−^ was observed with Pro-SISC-Me at pH > 7 (Figure 7a), while precipitate appeared in this pH range with SISC. In order to clarify this difference, UV-vis spectra were recorded for the Fe(III)—SISC (Figure S13a) and Fe(III)—Pro-SISC-Me (Figure 8a) systems at various pH values. In the first case, parallel to the formation of the species [Fe(III)L]^2+^ a band with λ_max_ = 610 nm appeared and shifted to the lower wavelengths (λ_max_ = 525 nm) as the bis complex was formed, while a strong absorbance decrease was seen at pH > 9 due to precipitation. In the Fe(III)—Pro-SISC-Me system, the formation of the mono complex was accompanied by the development of a band with λ_max_ = 600 nm, while λ_max_ was located at 490 nm as [Fe(III)L_2_]^+^ was formed, and this band increased up to pH 10.9, but λ_max_ practically remained constant, suggesting that the formation of [Fe(III)L_2_H_−1_] and [Fe(III)L_2_H_−2_]^−^ does not change the type of the coordinated donors. The absorbance was significantly decreased in the strongly basic pH range along with a blue shift, and most probably additional hydrolytic processes took place, e.g., formation of mixed hydroxido species, however for them we could not determine stability constants. The absorbance measured at 490 nm was constant between pH 4.8 and 8.0 (Figure 8a) where [Fe(III)L_2_]^+^ was formed, and it indicated a different coordination mode compared to the analogous SISC complex (525 nm). In addition, predominance curves calculated for the hypothetic Fe(III)—SISC—Pro-SISC-Me (1:2:2) system (Figure 7b) also exhibited clearly the stronger Fe(III) binding ability of Pro-SISC-Me over SISC, which might be also explained by an altered binding mode.

Further differences were observed when the redox behavior of the iron complexes was monitored by cyclic voltammetry. In the case of SISC, only a cathodic peak could be observed in the pH range of the formation of the Fe(III) complexes (not shown), unlike for Pro-SISC-Me (Figure 8b), where quasi-reversible processes could be seen in a wide pH range (3.9–10.8). The cathodic peak measured at pH 7.6 was located at much lower potential than in the case of the SISC complex (−0.59 V vs. −0.38 V, Figure S13b). Interestingly, the E_1/2_ value of the Fe(III)/Fe(II) redox couple for the Pro-SISC-Me complexes was −0.32 V vs. NHE, which was significantly lower as it was found for the STSC (E_1/2_ = −0.14 V vs. NHE) or Triapine (E_1/2_ = +0.08 V vs. NHE [34]) complexes. This low redox potential also indicated a strong preference of Pro-SISC-Me for Fe(III) over Fe(II), and it suggested the binding of harder Lewis-base donor atoms than would be expected from the (O,N,N) coordination mode, which was the case for the SISC complex. To reveal the coordination mode in the Fe(III)-Pro-SISC-Me complexes, crystallization experiments were performed and single crystals were obtained for the Na[Fe(III)(Pro-SISC-MeH_−2_)_2_](H_2_O)_4_ complex. 

**Figure 9:**
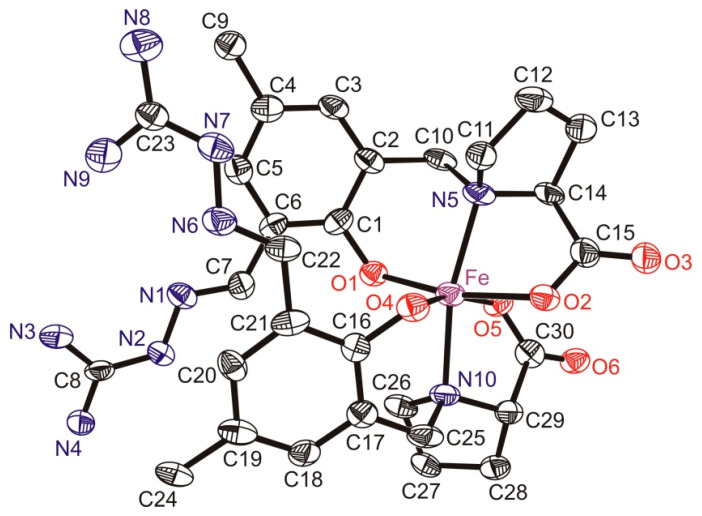
ORTEP view of Na[Fe(III)(Pro-SISC-MeH_−2_)_2_](H_2_O)_4_ (**2**) with thermal ellipsoids at 30% probability level. Water molecules, counter ion (Na^+^) and hydrogens are omitted for clarity. Selected bond length (Å) and angles (deg): Fe1-O1: 1.901(9); Fe1-O2: 2.057(9); Fe1-O4: 1.978(9); Fe1-O5: 2.100(9); Fe1-N10: 2.14(1); Fe1-N5: 2.14(1); O1-Fe1-O4: 98.9(4); O4-Fe1-O2: 87.1(4); O1-Fe1-O5: 91.9(4); O2-Fe1-O5: 84.5(4); O1-Fe1-O2: 167.6(4); O4-Fe1-O5: 164.5(4); N5-Fe1-N10: 164.2(5).

Molecular structure of the metal complex anion [Fe(III)(Pro-SISC-MeH_−2_)_2_]^−^ is shown in Figure 9 together with some selected bond lengths and angles. The Fe(III) has an elongated octahedral geometry with the tridentate coordination of two meridional ligands. The ligand has two possible coordination sites: the aminoguanidine and the proline part. In literature, there are two crystal structures of Fe(III) with pyridoxal-aminoguanidine (Reference code CELHIM and CELHOS) [35] where the guanidine coordination is realized. Surprisingly, in our case the proline part of the ligands coordinated with the metal ion by the phenolic oxygen, pyrrolidine nitrogen and one carboxylate oxygen. The two tridentate ligands coordinated in a way that the middle (nitrogen) donor atoms occupied the axial positions (Fe1-N5 and Fe1-N10 has somewhat longer bond lengths in comparison with the Fe-O bonds) so that the four oxygens defined the equatorial plane. Owing to the coupled chelate coordination, the octahedron was distorted and the O1-Fe1-O2 and O4-Fe1-O5 angles, similar to the N5-Fe1-N10 deviate from the theoretical 180°, as they were 167.6(4)°, 164.4(4)° and 164.1(5), respectively. The two phenyl rings turned to the same side of the complex and they were oriented almost parallel to each other (the angle between the ring planes is 8.0(7)° and the distance between the ring centroids was 4.973(9) Å. Due to the relatively large error in the collected data set, the guanidine =NH and –NH_2_ groups of the ligands were hardly distinguished, though shorter C-N distances (C8-N3 1.29(2) Å and C23-N9 1.30(2) Å were set to =NH and the longer ones (C8-N4 1.32(2) and C23-N8, 1.31(2) Å) were set to –NH_2_ groups. Even the exchange of the two groups in the crystal structure was possible. The crystal lattice was stabilized by C-H…O and N-H…O bonds between the neighboring ligands, and also through water molecules. The packing arrangement of crystal (**2**) from a, b and c crystallographic directions are shown in Figure S14. In all, the coordination of Pro-SISC-Me via the (O_carboxylate_^−^, N_proline_, O_phenolate_^−^) donor set can explain its different behavior in solution from the reference compound SISC. Most probably this is the binding mode in the [Fe(III)L_2_]^+^ complex, and [Fe(III)L_2_H_−1_] and [Fe(III)L_2_H_−2_]^−^ are formed by the deprotonation of the non-coordinating aminoguanidinium moieties. 

**Figure 10:**
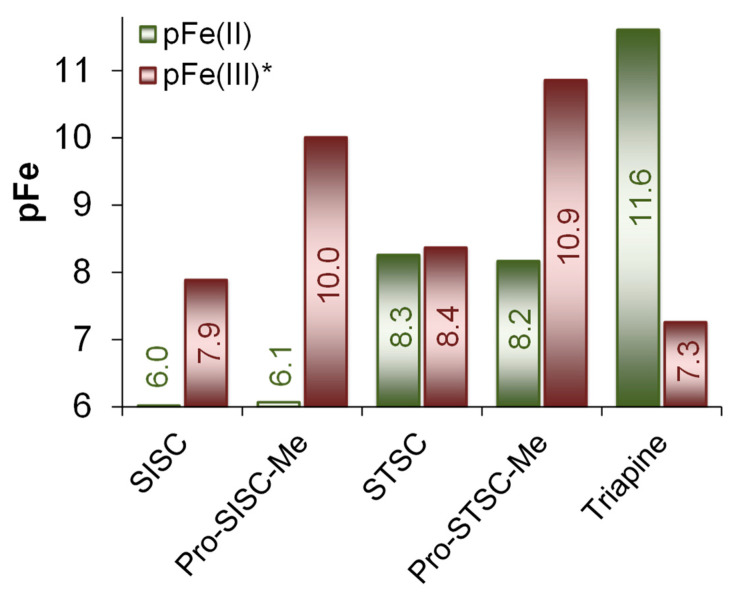
Fe(II)- and Fe(III)-binding affinity of SISC and Pro-SISC-Me compared to some related TSCs. pFe(II) (−log[Fe(II)]) and pFe(III)* (−logΣ[Fe(III)_i_(OH)_j_]) values calculated at pH 7.4 at *c*_Fe_ = 1 μM and *c*_ligand_ = 10 μM. ((30% (*v*/*v*) DMSO/H_2_O; *T* = 25 °C, *I* = 0.10 M (KCl)) Values for STSC, Pro-STSC-Me and Triapine complexes are calculated on the basis of the overall stability constants reported in Refs. [22,27,34].

The iron binding properties of SISC and Pro-SISC-Me were compared to structurally related salicylaldehyde TSCs (STSC, Pro-STSC-Me) and Triapine via the calculation of pFe values at pH 7.4 (Figure 10). Based on these values, the Fe(II)-binding ability of the ligands followed the order: SISC, Pro-SISC-Me < Pro-SSC-Me, STSC << Triapine, while the Fe(III)-binding of Triapine was the weakest. The proline hybrids had stronger Fe(III) binding than the reference compounds (SISC, STSC), however, the difference between the pFe(III) and pFe(II) values was the highest for Pro-SISC-Me, most likely due to its (O_carboxylate_^−^, N_proline_, O_phenolate_^−^) binding mode. Since it was reported that the iron preference of the TSCs can influence the cytotoxicity [15,20], the observed differences in the pFe values was expected to affect the bioactivity of these compounds as well. 

### 2.5. In Vitro Cytotoxicity of the Compounds

The cytotoxic activity of SISC and Pro-SISC-Me was assayed in the chemo-sensitive Colo-205, and the doxorubicin-resistant Colo-320 human colon adenocarcinoma cell lines, using the colorimetric 3-(4,5-dimethylthiazol-2-yl)-2,5-diphenyl-tetrazolium bromide (MTT) test. Additionally, the cytotoxicity was measured in normal human embryonal lung fibroblast cells (MRC-5). Determined IC_50_ values using 72 h incubation time are collected in Table 4. Measurements were also performed in the presence of one equivalent Cu(II) ions and comparative data for STSC and Triapine are also shown (Table 4). 

Based on the IC_50_ values, ligands SISC and Pro-SISC-Me are not cytotoxic, STSC has fairly weak cytotoxicity, and only Triapine is characterized by IC_50_ values in the low μM concentrations. The lower activity of SISC, Pro-SISC-Me and STSC can be explained by their inability to bind iron efficiently in both +2 and +3 oxidation states (see the pFe values in Figure 10), which seems to be necessary for biological activity and the iron complexes of these ligands cannot go through redox cycling between the two oxidation states. However, this is unlike Triapine, which acts as an efficient Fe(II) binder as well and is characterized by a moderate (and not too negative) redox potential (as is explained in the previous section).

In the presence of Cu(II) ions, SISC and STSC displayed more significant anticancer activity than the ligands alone, while the Triapine became less active. Pro-SISC-Me remained non-toxic upon complexation with Cu(II). The complex of STSC is neutral and lipophilic at pH 7.4 (logD_7.4_ = +1.14 [24]) which most probably accounts for the enhanced cytotoxic activity. The Cu(II) complex of SISC is more hydrophilic (logD_7.4_ = −0.49 ± 0.03) as it is present mostly in its positively charged form at pH 7.4 (90% [CuL]^+^, 10% [CuL(OH)]), and this feature can result in a smaller increase in the cytotoxicity, however, the complex is redox-active, based on our measurement. The Cu(II) complex of SISC was found to be cytotoxic against the human colon cancer cell line HCT16 as well as showing apoptosis induction [3].

In the case of Pro-SISC-Me, the [CuL]^+^ form also predominates, which contains the zwitter ionic (COO^−^, N_Pro_H^+^) moiety and as a result it is even more hydrophilic (logD_7.4_ = −0.87 ± 0.03), which is not advantageous for an efficient cellular uptake. The Triapine Cu(II) complex is also positively charged at pH 7.4 [21].

## 3. Materials and Methods

### 3.1. Chemicals

STSC, KCl, HCl, KOH, KNO_3_, DMSO, DMF, KSCN, tetrabutylammonium nitrate (TBAN), 4-(2-hydroxyethyl)-1-piperazineethanesulfonic acid (HEPES) and 4,4-dimethyl-4-silapentane-1-sulfonic acid (DSS) were purchased from Sigma-Aldrich (Budapest, Hungary) in puriss quality. The Fe(II) stock solution was obtained from fine Fe powder dissolved in a known amount of HCl solution under a purified, strictly oxygen-free argon atmosphere, then filtered, stored and used under anaerobic conditions. KSCN solution was used to check the absence of Fe(III) traces in the Fe(II) solution. The concentration of the Fe(II) stock solution was determined by permanganometric titrations under acidic conditions. FeCl_3_ and anhydrous CuCl_2_ were dissolved in known amounts of HCl and in water, respectively, in order to get the Fe(III) and Cu(II) stock solutions. Their concentrations were determined by complexometry, via the EDTA complexes. The strong acid content of the metal stock solutions were determined by pH-potentiometric titrations. All solvents were of analytical grade and used without further purification. Milli-Q water was used for sample preparation.

### 3.2. Synthesis of SISC and Pro-SISC-Me

SISC·HCl·0.5C_2_H_5_OH·H_2_O was prepared as reported in the literature [5]. For the preparation of Pro-SISC-Me·HCl·H_2_O, a solution of aminoguanidine bicarbonate (0.52 g, 3.8 mmol) in hot water (1 mL) treated with 2 equiv. 6 M HCl was added to a warm solution of the (2*S*)-1-[(3-formyl-2-hydroxy-5-methylphenyl)methyl]-pyrrolidine-2-carboxylic acid (1.00 g, 3.8 mmol) in methanol (5 mL). The reaction mixture was stirred at 75 °C for 2 h. After cooling the solution to room temperature, methanol was removed under reduced pressure and water (2 mL) was added. The white precipitate was filtered off, washed with water and dried in vacuo overnight. Yield: 0.91 g (61%). Calcd. for C_15_H_22_N_5_O_3_Cl·2H_2_O (M_r_ 391.85), %: C, 45.98; H, 6.69; 17.87; Cl, 9.05. Found, %: C, 45.87; H, 6.54; N, 17.57; Cl, 9.24. 

### 3.3. X-ray Data Collection, Structure Solution and Refinement for Pro-SISC-Me·HBr·H_2_O (**1**) and the Complex Na[Fe(III)(Pro-SISC-MeH_−2_)_2_](H_2_O)_4_ (**2**)

Single crystals suitable for X-ray diffraction experiment of **1** were picked up from the synthesized product, while single crystals of **2** were grown from methanol. The solution contained FeCl_3_ and Pro-SISC-Me at 1:2 metal-to-ligand ratio and NaOH was added step by step until the green solution turned into red. A red, single crystal was mounted on a loop and transferred to the goniometer.

X-ray diffraction data for **1** and **2** were collected on a Bruker X8 APEXII CCD and Rigaku RAXIS-RAPID II diffractometer, respectively. The data were processed using SAINT software [36] for **1** and CrystalClear software [37] for **2**. Crystal data, data collection parameters, and structure refinement details for **1** and **2** are given in Tables S3 and S4, respectively. Both structures were solved by direct methods and refined by full-matrix least-squares techniques. Non-hydrogen atoms were refined with anisotropic displacement parameters. H atoms were inserted into calculated positions and refined with a riding model, even though their positions could be determined in the case of **1** by a difference Fourier map.

The following computer programs and hardware were used for **1**: structure solution: SHELXS; refinement: SHELXL [38]; molecular diagrams: ORTEP [39]; computer: Intel CoreDuo. In the case of **2**, Sir2014 [40] and SHELXL [38] under WinGX [41] software were used for structure solution and refinement, respectively. Due to the poor crystal quality and small crystal size of **2**, high R values were obtained which prevented the determination of hydrogen atomic positions from the difference Fourier map. Therefore, hydrogen atoms were included in the structure factor calculations, but they were not refined. The isotropic displacement parameters of the hydrogen atoms were approximated from the U(eq) value of the atom they were bonded to. Water hydrogens were fixed in geometric positions using DFIX and DANG options to obtain reasonable positions for them. Selected bond lengths and angles of compounds were calculated by PLATON software [42].

The crystallographic data files for the compounds have been deposited with the Cambridge Crystallographic Database as CCDC 2143435 (**1**) and CCDC 2144192 (**2**). 

### 3.4. pH-Potentiometry

The pH-potentiometric measurements for the determination of the proton dissociation constants of the ligands and the overall stability constants of the metal complexes were carried out at 25.0 ± 0.1 °C in water or in 30% (*v*/*v*) DMSO/H_2_O as solvent and at an ionic strength of 0.10 M (KCl). The titrations were performed with carbonate-free KOH solution of known concentration (0.10 M). The concentrations of the base and the HCl were determined by pH-potentiometric titrations. An Orion 710A pH-meter equipped with a Metrohm combined electrode (type 6.0234.100), and a Metrohm 665 Dosimat burette were used for the titrations. The electrode system was calibrated to the pH = −log[H+] scale by means of blank titrations (strong acid vs. strong base: HCl vs. KOH) according to the method suggested by Irving et al. [43]. The average water ionization constant pK_w_ was 13.76 ± 0.05 (water) or 14.52 ± 0.05 (30% (*v*/*v*) DMSO/H_2_O). The pH-potentiometric titrations were performed in the pH range 2.0–12.5. The initial volume of the samples was 10.0 mL. The ligand concentration was 2 mM and metal ion-to-ligand ratios of 1:1–1:3 were used. The accepted fitting of the titration curves was always less than 0.01 mL. Argon was always passed over the solutions during the titrations. The exact concentration of the ligand stock solutions together with the proton dissociation constants were determined by pH-potentiometric titrations with the use of the computer program HYPERQUAD [44]. It was also utilized to establish the stoichiometry of the complexes and to calculate the stability constants (β(M_p_L_q_H_r_)). β(M_p_L_q_H_r_) is defined for the general equilibrium pM + qL + rH ⇋ M_p_L_q_H_r_ as β(M_p_L_q_H_r_) = [M_p_L_q_H_r_]/[M]^p^[L]^q^[H]^r^, where M denotes the metal ion and L the completely deprotonated ligand. In all calculations exclusively, titration data were used from experiments in which no precipitate was visible in the reaction mixture.

### 3.5. UV-Vis Spectrophotometry and Fluorometry

An Agilent Cary 8454 diode array spectrophotometer (Agilent Technologies, Santa Clara, CA, USA) was used to record the UV-vis spectra at an interval of 200–800 nm. The path length was 1 or 2 cm. Spectrophotometric titrations were performed in water or in 30% (*v*/*v*) DMSO/H_2_O on samples containing the ligands at 50 μM–1 mM concentration, in the pH range from 1.0 to 12.5 in the absence or in the presence of 1 or 0.5 or 0.33 equiv. metal ions. K_a_ values of the ligands and the UV-vis spectra of the individual species were calculated by the computer program PSEQUAD [45].

The redox reaction of the Cu(II) complexes with GSH and ascorbic acid was studied in 30% (*v*/*v*) DMSO/H_2_O at 25.0 ± 0.1 °C using a special, tightly closed tandem cuvette (Hellma Tandem Cell, 238-QS (Hellma Materials GmbH, Jena, Germany)). The reactants were separated until the reaction was triggered. Both isolated pockets of the cuvette were completely deoxygenated by bubbling of a stream of Ar for 10 min before mixing the reactants. Spectra were recorded before and then immediately after the mixing, and changes were followed until no further absorbance change was observed. One of the isolated pockets contained the reducing agent, while the other contained the metal complex, and their final concentrations after mixing were 250 μM and 25 μM, respectively. The pH of all the solutions was adjusted to 7.40 by 50 mM HEPES buffer and an ionic strength of 0.1 M (KCl) was applied. The stock solutions of the reducing agents and the complexes were freshly prepared every day.

Distribution coefficients (D_7.4_) values were determined by the traditional shake-flask method in *n*-octanol/buffered aqueous solution at pH 7.40 (20 mM HEPES, 0.10 M KCl) at 25.0 ± 0.2 °C as described previously [20,24]. 

The fluorescence spectra of the ligands were recorded on a Hitachi F-4500 spectrofluorometer in water at pH 7.4 (Hitachi High-Technologies Corporation, Tokyo, Japan) using a 1 × 1 cm quartz cell. 

### 3.6. ^1^H NMR Spectroscopic Titrations

The ^1^H NMR studies for the ligands were carried out on a Bruker Ultrashield 500 Plus instrument (Billerica, MA, USA). DSS was used as an internal NMR standard and WATERGATE water suppression pulse scheme was used. Spectra were recorded in 10% (*v*/*v*) D_2_O/H_2_O or in 30% (*v*/*v*) DMSO-d_6_/H_2_O solvent mixtures in a concentration of 1 mM, respectively, at ionic strength of 0.10 M (KCl).

### 3.7. EPR Spectroscopy

All EPR spectra were recorded with a BRUKER EleXsys E500 spectrometer (microwave frequency 9.54 GHz, microwave power 13 mW, modulation amplitude 5 G, modulation frequency 100 kHz, Bruker BioSpin Corporation, Billerica, MA, USA) at 77 K for the Cu(II)—SISC complexes and at room temperature for the Cu(II)—Pro-SISC-Me species. EPR spectra were recorded at different pH values at two metal-to-ligand concentration ratios: 0.25 mM Cu(II) and 0.25 mM or 0.50 mM SISC in 30% DMSO/water solution, and 1.0 mM Pro-SISC-Me and 1.0 mM or 0.5 mM Cu(II) in water. The ionic strength was adjusted with KCl (*I* = 0.1 M). KOH solution was added to the stock solution to change the pH between pH = 2.5–12.5, which was measured with a Radiometer PHM240 pH/ion Meter equipped with a Metrohm 6.0234.100 glass electrode. The isotropic EPR spectra were recorded at room temperature in a circulating system, and a Heidolph Pumpdrive 5101 peristaltic pump was used to circulate the solution from the titration vessel through a capillary tube into the cavity of the instrument. For the anisotropic EPR spectra, a pH titration was performed outside of the cavity and at selected pH values, and 200 μL samples were transferred from the titration vessel into EPR tubes and the spectra were recorded in a Dewar containing liquid nitrogen.

The anisotropic EPR spectra were analyzed with the EPR program [46]. Rhombic g-tensor (g_x,_ g_y_, g_z_) and copper hyperfine tensor (A_x_, A_y_, A_z_) have been used. The nitrogen superhyperfine structure was taken into account with a rhombic hyperfine tensor (a_x_^N^, a_y_^N^, a_z_^N^), where the xyz directions referred to the g-tensor orientations. Orientation-dependent linewidth parameters (α, β, and γ) were used to fit the linewidths through the equation σ_MI_ = α + βM_I_ + γM_I_^2^, where M_I_ denotes the magnetic quantum number of Cu(II) ion. Since natural CuCl_2_ was used for the measurements, the spectra were calculated by the summation of spectra ^63^Cu and ^65^Cu, weighted by their natural abundances. The hyperfine and superhyperfine coupling constants and the relaxation parameters were obtained in field units (Gauss = 10^−4^ T). The series of room temperature EPR spectra were simulated simultaneously by the ‘two-dimensional’ method using the 2D_EPR program [47]. Each component curve was described by the isotropic EPR parameters g_0_, A_0_ copper hyperfine and A_0_^N^ nitrogen hyperfine couplings and isotropic linewidth parameters (α, β, and γ). The concentrations of the complexes were varied by fitting their formation constants β(M_p_L_q_H_r_). The details of the analysis were published previously [20,22,27,30].

### 3.8. Cyclic Voltammetry

Cyclic voltammograms of the Cu(II) and Fe(III) complexes in 30% (*v*/*v*) DMSO/H_2_O containing 1 mM Cu(NO_3_)_2_ or 1 mM or 0.5 mM FeCl_3_ and 1 mM ligand were recorded at 25.0 ± 0.1 °C. Ionic strength was 0.10 M (TBAN). Measurements were performed on a conventional three-electrode system under nitrogen atmosphere using an Autolab PGSTAT 204 potentiostat/galvanostat monitored by Metrohm’s Nova software (Metrohm Autolab B.V., Utrecht, The Netherlands). Samples were purged with argon for 15 min before recording the cyclic voltammograms. Platinum electrodes were used as the working and auxiliary electrode and Ag/AgCl/3 M KCl as a reference electrode. The electrochemical system was calibrated with an aqueous solution of K_3_[Fe(CN)_6_] (E_1/2_ = +0.386 V vs. NHE).

### 3.9. In Vitro Cell Studies: Cell Lines and Culture Conditions and MTT Assay

All cell culture reagents were obtained from Sigma-Aldrich and plastic ware from Sarstedt (Nümbrecht, Germany). Human colon Colo-205 (chemo-sensitive) and Colo-320 (doxorubicin-resistant) adenocarcinoma and MRC-5 human embryonal lung fibroblast cell lines were purchased from LGC Promochem, Teddington, UK. The colon adenocarcinoma cells were cultured in Roswell Park Memorial Institute (RPMI) 1640 medium supplemented with 10% heat-inactivated fetal bovine serum, 2 mM L-glutamine, 1 mM sodium pyruvate and 100 mM HEPES. The MRC-5 cell line was cultured in Eagle’s Minimal Essential Medium (EMEM, Sigma-Aldrich, St. Louis, MO, USA) supplemented with a non-essential amino acid (NEAA) mixture (Sigma-Aldrich, St. Louis, MO, USA) a selection of vitamins, 10% heat-inactivated foetal bovine serum (FBS), 2 mM L-glutamine (Sigma-Aldrich, St. Louis, MO, USA), 1 mM Na-pyruvate (Sigma-Aldrich, St. Louis, MO, USA), nystatin (Sigma-Aldrich, St. Louis, MO, USA) and a penicillin-streptomycin mixture (Sigma-Aldrich, St. Louis, MO, USA) in concentrations of 100 U/L and 10 mg/L, respectively. The cells were incubated at 37 °C, in a 5% CO_2_, 95% air atmosphere. All cell lines were detached with Trypsin-Versene^®^ (EDTA) solution for 5 min at 37 °C.

The tested compounds were dissolved in 90% (*v*/*v*) DMSO/H_2_O using 8 mM concentration, and the and in the final samples the DMSO content was always lower than 1%. Then stock solutions were diluted in complete culture medium, and two-fold serial dilutions of compounds were prepared in 100 μL of the medium, horizontally. The semi-adherent colon adenocarcinoma cells were treated with Trypsin-Versene (EDTA) solution. They were adjusted to a density of 1 × 10^4^ cells in 100 μL of RPMI 1640 or EMEM medium and were added to each well, with the exception of the medium control wells. The final volume of the wells containing compounds and cells was 200 μL. The plates containing Colo-205, Colo-320 or MRC-5 cells were incubated at 37 °C for 72 h; at the end of the incubation period, 20 μL of MTT solution (from a stock solution of 5 mg/mL) were added to each well. After incubation at 37 °C for 4 h, 100 μL of SDS solution (10% in 0.01 M HCI) were added to each well, and the plates were further incubated at 37 °C overnight. Cell growth was determined by measuring the optical density (OD) at 540/630 nm with a Multiskan EX plate reader (Thermo Labsystems, Cheshire, WA, USA). Inhibition of the cell growth (expressed as IC_50_: inhibitory concentration that reduces by 50% the growth of the cells exposed to the tested compounds) was determined from the sigmoid curve where 100 − ((OD_sample_ − OD_medium control_)/(OD_cell control_ − OD_medium control_)) × 100 values were plotted against the logarithm of compound concentrations. Curves were fitted by GraphPad Prism software (2021, Graphpad Software, San Diego, CA, USA) [48] using the sigmoidal dose–response model (comparing variable and fixed slopes). The IC_50_ values were obtained from at least three independent experiments.

## 4. Conclusions

Two salicylaldehyde aminoguanidine derivatives (SISC, Pro-SISC-Me) were prepared as salicylaldehyde thiosemicarbazone analogues to investigate the effect of the exchange of the thioamide sulfur (=S) to an iminosemicarbazone (=NH) moiety on the pK_a_ values and lipophilic character, the complex formation ability with endogenous metal ions such as Cu(II), Fe(II) and Fe(III) and their cytotoxic activity. Pro-SISC-Me contains an additional L-proline moiety to provide enhanced water solubility due to its zwitter ionic structure.

The proton dissociation processes were characterized by the combined use of pH-potentiometry, UV-vis and ^1^H NMR spectroscopic titrations in 30% (*v*/*v*) DMSO/H_2_O and in water. SISC was characterized by two pK_a_ values which belong to the overlapping deprotonation processes of the phenolic-OH and the positively charged aminoguanidinium (C(=NH_2_^+^)NH_2_) functional groups. Pro-SISC-Me has the additional pK_a_ of the proline carboxylic acid group, while the proline nitrogen remains protonated in the whole pH range studied. Based on the pK_a_ values, SISC is found at pH 7.4 in its H_2_L^+^ (64%) and HL (36%) forms, while the analogous STSC is neutral (H_2_L), and Pro-SISC-Me is present in its H_2_L^+^ (33%) and HL (67%) forms which contain the zwitter ionic (N_Pro_H^+^, COO^−^) amino acid residue contributing to its considerably hydrophilic character. 

The stability of the Cu(II) complexes was characterized by pH-potentiometry, and the speciation model was confirmed by UV-vis measurements. Mono-ligand complexes ([CuL]^+^, [CuLH_−1_], [CuLH_−2_]^−^) were found exclusively even at ligand excess. The Cu(II) complexes of both SISC and Pro-SISC-Me possess high stability in solution, which is comparable to that of the related thiosemicarbazones: STSC and Pro-STSC-Me. Complex [CuL]^+^ predominates at pH 7.4 in which the ligands coordinate in a tridentate mode via the phenolato oxygen, azomethine-N and one of the guanidine nitrogens based on the EPR spectroscopic parameters. Complexes [CuLH_−1_] and [CuLH_−2_]^−^ are mixed hydroxido species. The EPR spectroscopic data suggest the same coordination modes in the corresponding SISC and Pro-SISC-Me complexes. The [CuL]^+^ complexes could not be reduced by ascorbate at pH 7.4, whereas they could oxidize GSH efficiently. 

Unlike TSCs, SISC and Pro-SISC form mono complexes with Fe(II) exclusively under the applied conditions, however their stability, which is otherwise similar to each other, is much lower compared to that of the Cu(II) complexes. On the contrary, bis complexes are also formed with Fe(III) ions and complex [Fe(III)L_2_]^+^ is found at pH 7.4 in solution. Interestingly, [Fe(III)L_2_]^+^ formed with Pro-SISC-Me has significantly higher stability in comparison to the SISC complex. Complexes [Fe(III)L_2_]^+^ have different structures also suggested by their different λ_max_ (Pro-SISC-Me: 490 nm, SISC: 525 nm) and redox potential (Pro-SISC-Me: E_1/2_ = −0.32 V vs. NHE, SISC: only a cathodic peak at −0.17 V vs. NHE). In the case of Pro-SISC-Me, the proline moiety is involved in the coordination and the ligands bind in the bis complex via the (O_carboxylate_^−^, N_Pro_, O_phenolate_^−^) donor set confirmed by X-ray crystallographic analysis. Fe(III) ions form complexes that possess higher stability with both ligands than Fe(II), although, the (O_carboxylate_^−^, N_Pro_, O_phenolate_^−^) binding mode brings the even stronger Fe(III) preference over Fe(II) of Pro-SISC-Me compared to SISC. Most likely, this feature contributes to the lack of cytotoxic activity of SISC and Pro-SISC. It is worth noting that the redox active Cu(II) complex of SISC displayed a higher cytotoxic activity than the ligand alone, whereas the Cu(II) complex of Pro-SISC-Me was inactive, most probably due to its fairly hydrophilic nature.

## Figures and Tables

**Table 1:** pK_a_ values of SISC and Pro-SISC-Me determined by various methods in water and in 30% (*v*/*v*) DMSO/H_2_O, their *n*-octanol/water distribution coefficients (logD_7_._4_) and fluorescence λ_EX_(max) and λ_EM_(max) values at pH 7.4 in water. HL is the neutral form of the ligands. (*T* = 25 °C, *I* = 0.10 M (KCl)).

		SISC		Pro-SISC-Me		
Method	Medium	pK_1_ (H_2_L^+^)	pK_2_ (HL)	pK_1_ (H_3_L^2+^)	pK_2_ (H_2_L^+^)	pK_3_ (HL)
pH-pot. ^a^	H_2_O	7.64 ± 0.09	9.96 ± 0.09	2.52 ± 0.08	7.25 ± 0.06	9.61 ± 0.05
UV-vis		7.64 ± 0.03	10.12 ± 0.03	n.d. ^b^	7.30 ± 0.03	9.67 ± 0.03
^1^H NMR		-	-	≤2.5 ^c^	7.25 ± 0.03 ^c^	9.75 ± 0.03 ^c^
pH-pot. ^a^	30% DMSO/H_2_O	7.62 ± 0.03	10.63 ± 0.02	1.80 ± 0.08	7.08 ± 0.02	9.91 ± 0.02
UV-vis		7.56 ± 0.03	10.67 ± 0.03	n.d. ^b^	7.07 ± 0.03	9.84 ± 0.03
^1^H NMR		7.68 ± 0.03	10.72 ± 0.03	-	-	-
logD_7.4_	*n*-octanol/H_2_O	+0.83 ± 0.03		−0.88 ± 0.05		
λ_EX(max)_	H_2_O	285 nm, 324 nm		286 nm, 378 nm		
λ_EM(max)_	H_2_O	480 nm		495 nm		

^a^ pH-pot. = pH-potentiometry; ^b^ The deprotonation of COOH does not result in spectral changes in the UV-vis wavelength range, thus pK_a_ could not be determined by this method; ^c^ These pK_a_ values belong to the major species (E isomer), while pK_a_ = 7.55 ± 0.02 was determined for the minor (Z) isomer and no other pK_a_ could be determined as spectral changes were not detected between pH 9.3 and 11.5.

**Table 2:** Overall stability constants (β) of the Cu(II) complexes formed with SISC and Pro-SISC-Me determined by pH-potentiometry in 30% (*v*/*v*) DMSO/H_2_O and in water in addition to pCu(II) (−log[Cu(II)]) values calculated at pH 7.4 at *c*_Cu(II)_ = 1 μM and *c*_ligand_ = 10 μM. HL is the neutral form of the ligands. (*T* = 25 °C, *I* = 0.10 M (KCl)).

	SISC	Pro-SISC-Me	Pro-SISC-Me
	30% DMSO	30% DMSO	H_2_O
logβ [CuL]^+^	14.74 ± 0.02	15.08 ± 0.02	14.99 ± 0.02
logβ [CuLH_−1_]	6.28 ± 0.07	6.57 ± 0.05	6.96 ± 0.03
logβ [CuLH_−2_]^−^	-	−4.42 ± 0.06	−3.22 ± 0.04
pCu(II) ^a^	12.08	13.39	13.59

^a^ pCu(II) for STSC: 13.28 and Pro-STSC-Me: 13.40. Calculated on the basis of the overall stability constants reported in Refs. [22,27].

**Table 3:** Overall stability constants (β) of the Fe(II) and Fe(III) complexes formed with SISC and Pro-SISC-Me determined by pH-potentiometry in 30% (*v*/*v*) DMSO/H_2_O. HL is the neutral form of the ligands. (*T* = 25 °C, *I* = 0.10 M (KCl)).

	SISC	Pro-SISC-Me
logβ [Fe(II)L]^+^	7.20 ± 0.06	6.83 ± 0.04
logβ [Fe(II)LH_−1_]	−0.73 ± 0.07	−1.08 ± 0.05
logβ [Fe(II)LH_−2_]^−^	−8.94 ± 0.08	<−11
logβ [Fe(III)L]^2+^	14.96 ± 0.05	15.25 ± 0.04
logβ [Fe(III)L_2_]^+^	27.46 ± 0.05	27.63 ± 0.05
logβ [Fe(III)L_2_H_−1_]	-	18.40 ± 0.07
logβ [Fe(III)L_2_H_−2_]^−^	-	8.64 ± 0.08

**Table 4:** In vitro cytotoxicity of SISC, Pro-SISC-Me, STSC and Triapine, their Cu(II) complexes formed at 1:1 metal-to-ligand ratio, and CuCl_2_ expressed as IC_50_ values in Colo-205 and Colo-320 human colon adenocarcinoma and MRC-5 human lung fibroblast cells. Exposition time: 72 h.

IC_50_/μM	Colo-205	Colo-320	MRC-5
SISC	>80	>80	>80
Cu(II)—SISC	18.7 ± 1.2	34.0 ± 7.6	58.9 ± 2.1
Pro-SISC-Me	>80	>80	>80
Cu(II)—Pro-SISC-Me	>80	>80	>80
STSC ^a^	69.7 ± 1.9	52.2 ± 6.8	88 ± 19
Cu(II)—STSC ^a^	0.89 ± 0.09	0.64 ± 0.01	0.65 ± 0.10
Triapine ^a^	3.28 ± 0.63	2.17 ± 0.15	31.4 ± 9.2
Cu(II)—Triapine ^a^	11.4 ± 1.4	26.00 ± 0.73	35.61 ± 0.69
CuCl_2_	44.1 ± 0.4	48.7 ± 0.4	43.9 ± 0.3

^a^ Data are taken from Ref. [26].

## Data Availability

Not applicable.

## Supplementary Materials

The following supporting information can be downloaded at: https://www.mdpi.com/article/10.3390/molecules27072044/s1, Figure S1: UV-vis spectra of SISC; Figure S2: UV-vis spectra of Pro-SISC-Me; Figure S3: ^1^H NMR spectra of SISC; Figure S4: ^1^H NMR spectra of Pro-SISC-Me; Figure S5: UV-vis spectra of the ligands due to the partitioning experiments; Figure S6: Fluorescence spectra of the ligands; Figure S7: pH-potentiometric titration curves for the Cu(II)—ligand systems; Figure S8: UV-vis absorption spectra of the Cu(II)—SISC system; Figure S9: UV-vis absorption spectra of the Cu(II)—Pro-SISC-Me system; Figure S10: EPR spectra of the Cu(II)—SISC system at 77 K; Figure S11: Cyclic voltammograms recorded for Cu(II)—SISC system; Figure S12: pH-potentiometric titration curves for the Fe(II)—SISC system and concentration distribution curves for the Fe(II) complexes; Figure S13: UV-vis absorption spectra of the Fe(III)—SISC system and cyclic voltammograms of the iron complexes at pH 7.6; Figure S14: Packing arrangement of crystal (**2**); Table S1: Isotropic EPR parameters for the Cu(II)-Pro-SISC-Me complexes; Table S2: Anisotropic EPR parameters for the Cu(II)-SISC complexes; Tables S3 and S4: Crystal data and structure refinement of crystal (**1**) and (**2**).

## Sample Availability

Samples of the compounds are available from the authors.
