# Supplementary material for: Solution Equilibrium Studies on Salicylidene Aminoguanidine Schiff Base Metal Complexes: Impact of the Hybridization with L-Proline on Stability, Redox Activity and Cytotoxicity
Source: Molecules. 2022 Mar 22;27(7):2044. doi: 10.3390/molecules27072044 (PMC9000575; doi:10.3390/molecules27072044)
Supplement: Supplementary file 1 [file molecules-27-02044-s001.zip › molecules-1638095-supplementary.pdf]

Supporting Information for

Solution equilibrium studies on salicylidene aminoguanidine Schiff base metal complexes: impact of the hybridization with L-proline on stability, redox activity and cytotoxicity

Orsolya Dömötör, Nóra V. May, G. Tamás Gál, Gabriella Spengler, Aliona Dobrova, Vladimir B. Arion and Éva A. Enyedy*

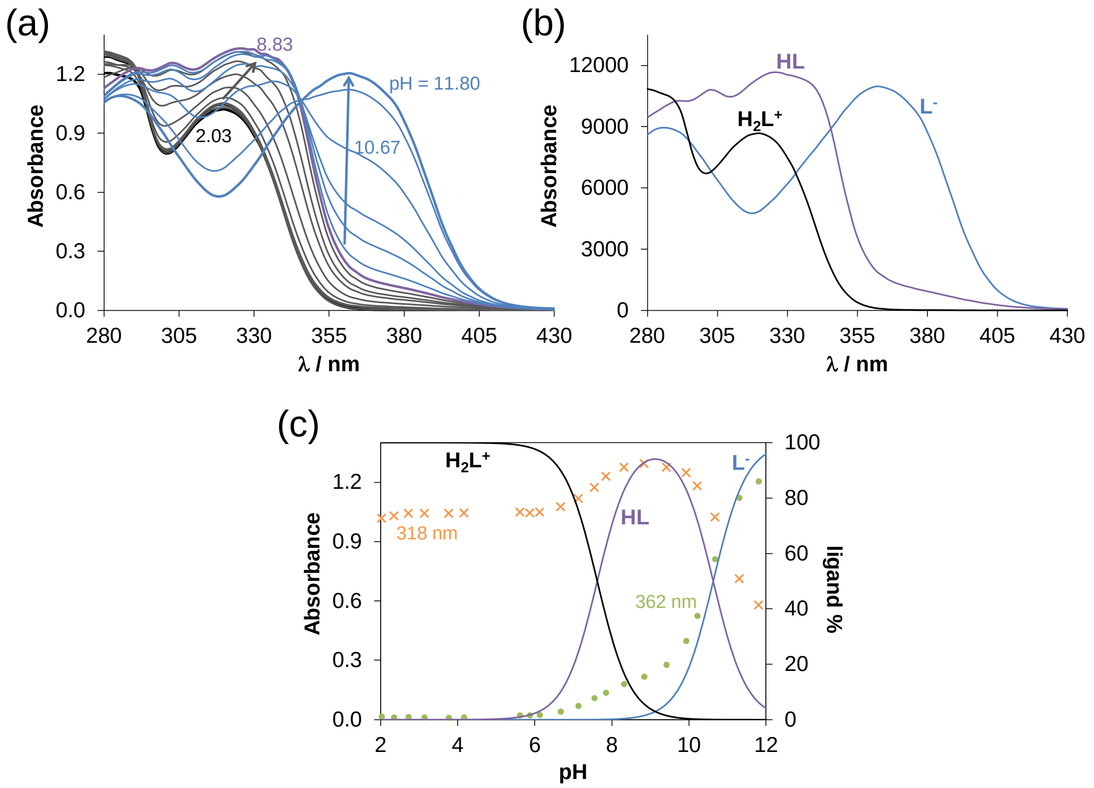

Figure S1. (**a**) UV-vis absorption spectra of SISC recorded in 30% (*v*/*v*) DMSO/H_2O at various pH values, and (**b**) individual molar absorbance spectra of the ligand species in the different protonation states. (**c**) Concentration distribution curves with absorbance values measured at 318 nm (×) and 362 nm (●). {c_{ligand} = 120 μM; T = 25 ˚C; I = 0.10 M (KCl); ℓ = 1 cm}

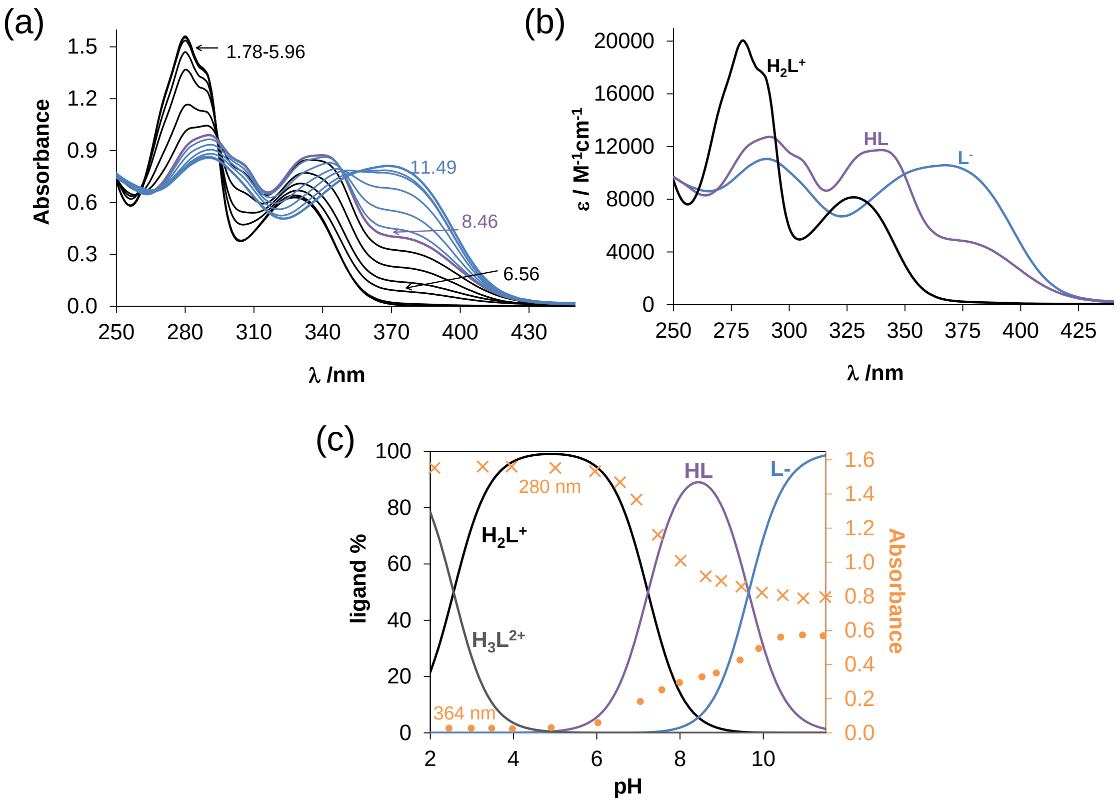

Figure S2. (**a**) UV-vis absorption spectra of Pro-SISC-Me recorded at various pH values in water. (**b**) Individual molar absorbance spectra of the ligand species in the different protonation states. (**c**) Concentration distribution curves with absorbance values measured at 280 nm (×) and 364 nm (●). {c_{ligand} = 73 μM; T = 25 °C; I = 0.10 M (KCl); ℓ = 1 cm}

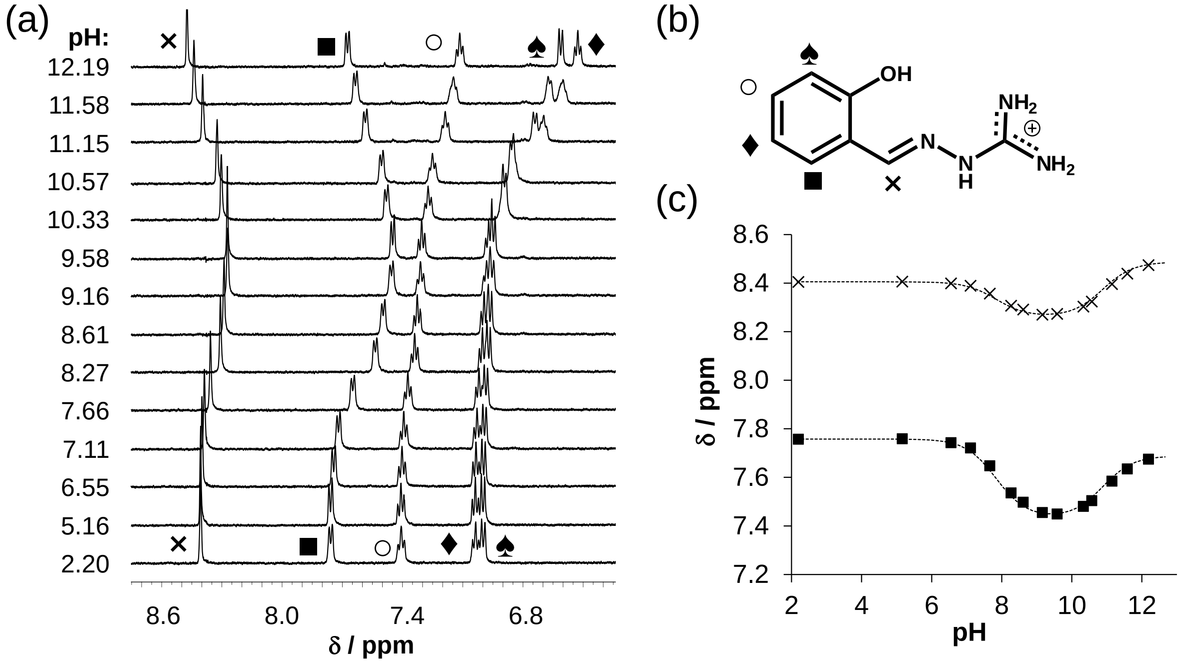

Figure S3. (**a**) ^{1}H NMR spectra of SISC recorded at the indicated pH values, (**b**) notation of the symbols at the various peaks, and (**c**) pH-dependent chemical shifts of two selected CH protons (×, ■) with the fitted curves (dashed lines). {c_{ligand} = 1.0 mM; 30% (*v*/*v*) d_6-DMSO/H_2O; pH = 2.2 – 12.2; *T* = 25 °C; *I* = 0.10 M (KCl)}.

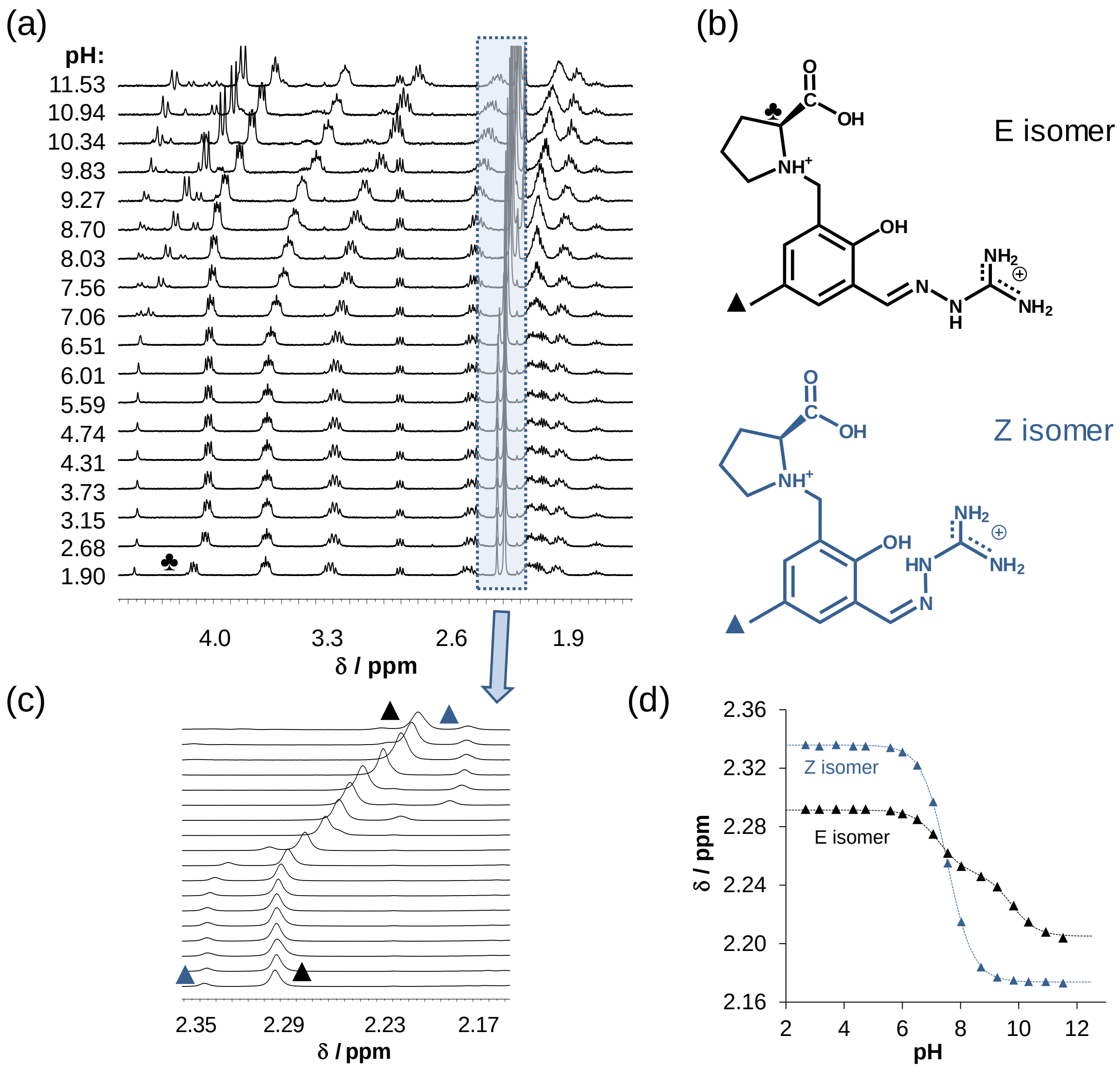

Figure S4. (**a**) ^{1}H NMR spectra of Pro-SISC-Me in the high field region recorded at the indicated pH values, (**b**) structure of the E and the Z isomers with the notation of the symbols of the CH_3 protons (▲,▲), and (**c**) ^{1}H NMR spectra in the chemical shift range of the CH_3 protons, and (**d**) the chemical shifts of the CH_3 protons plotted against the pH for the E isomer (▲) and the Z isomer (▲) with the fitted curves (dashed lines). {c_{ligand} = 1.0 mM; 10% (*v*/*v*) D_2O/H_2O; pH = 2.2 – 12.2; *T* = 25 °C; *I* = 0.10 M (KCl)}.

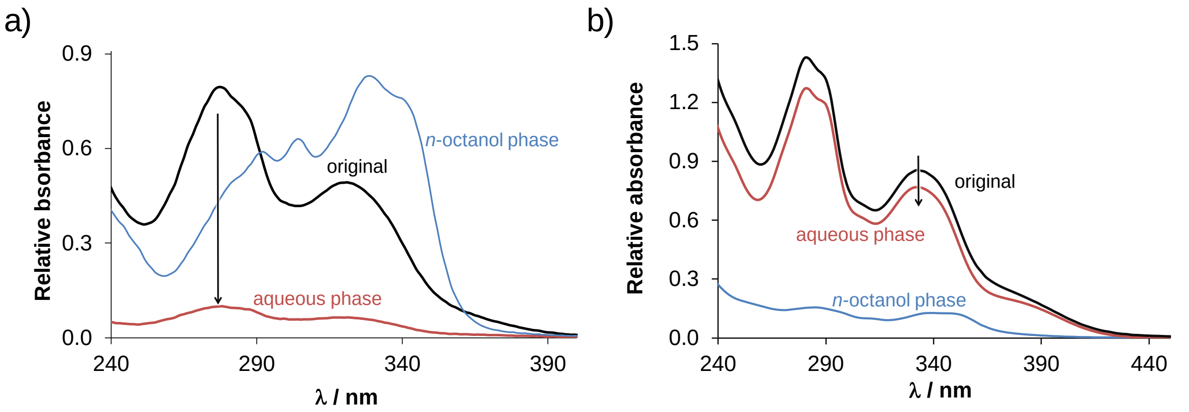

Figure S5. UV-vis absorption spectra of (**a**) SISC and (**b**) Pro-SISC-Me recorded for the original solution (black line) and for the aqueous (red line) and *n*-octanol (blue line) phases after partitioning. {pH = 7.4 (10 mM HEPES); *I* = 0.1 M (KCl); *T* = 25 °C}.

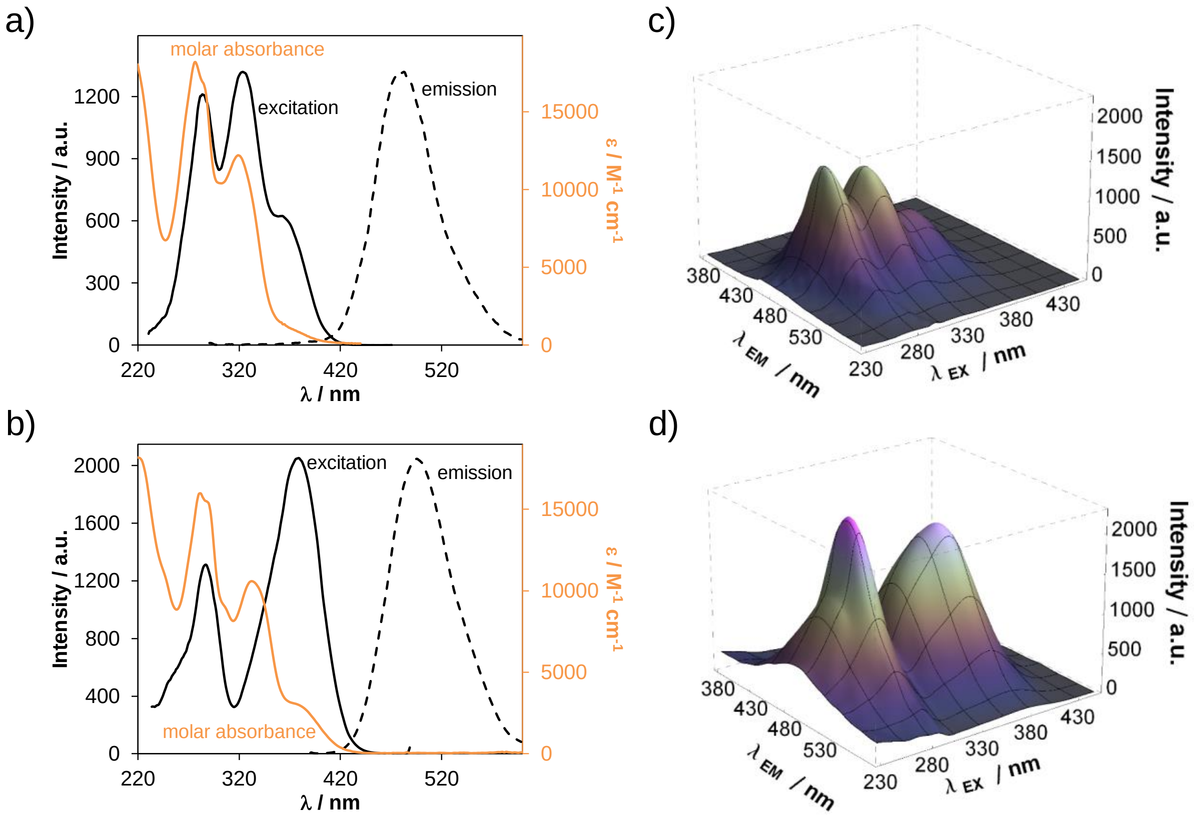

Figure S6. Molar UV-vis absorbance (orange line), excitation (black solid line) and emission (black dashed line) spectra of (**a**) SISC, and (**b**) Pro-SISC-Me, and the 3-dimentional fluorescence spectrum of (**c**) SISC and (**d**) Pro-SISC-Me at pH 7.40 in water. {c_{ligand} = 10 μM; *T* = 25 ˚C; *I* = 0.10 M (KCl); *ℓ* = 1 cm}

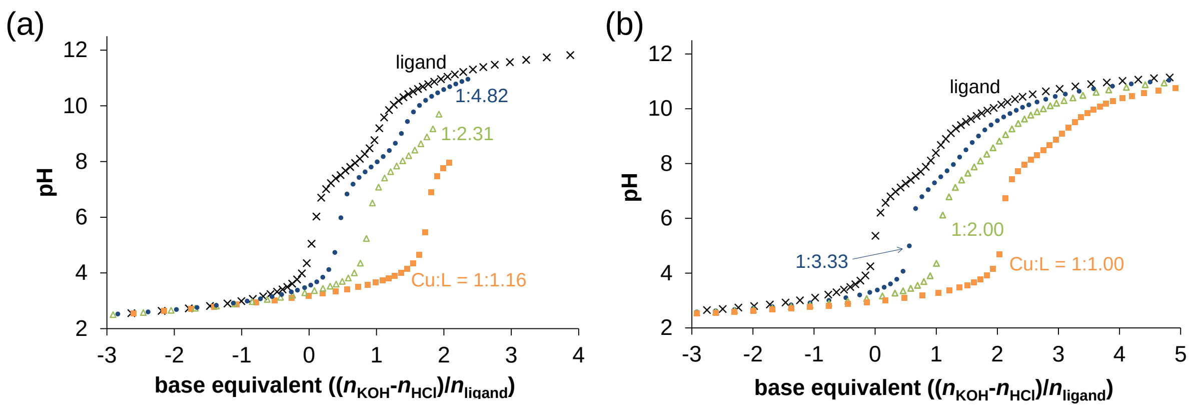

Figure S7. pH-potentiometric titration curves obtained for the (**a**) Cu(II) – SISC system in 30% (v/v) DMSO/H_2O and (**b**) Cu(II) – Pro-SISC-Me system in water at various metal-to-ligand ratios (indicated in the figure). {c_{ligand} = 1 mM; T = 25 ˚C; I = 0.10 M (KCl)}

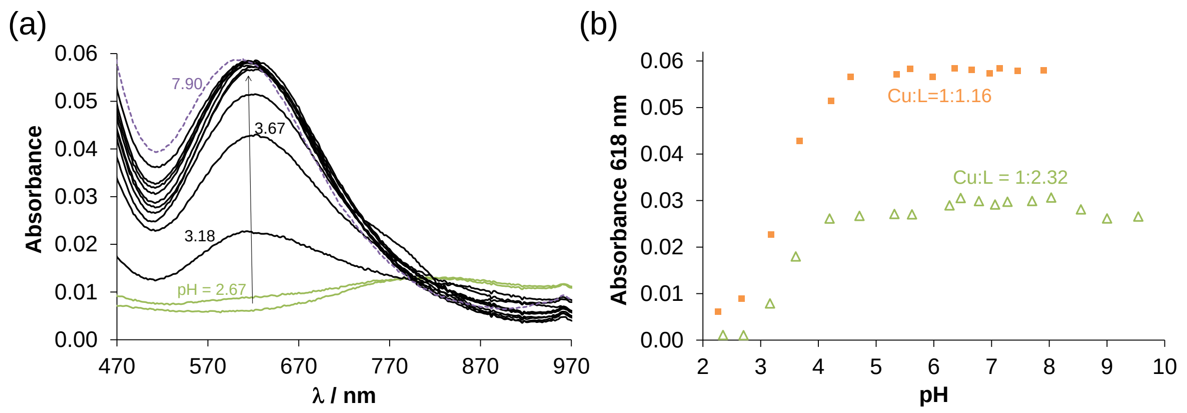

Figure S8. (**a**) UV-vis absorption spectra of the Cu(II) – SISC (1:1.16) system recorded at various pH values and (**b**) changes of the absorbance at 618 nm at 1:1.16 and 1:2.32 Cu(II)-to-ligand ratios. {30% (*v*/*v*) DMSO/H_2O; c_{ligand} = 564 μM; T = 25 ˚C; I = 0.10 M (KCl); ℓ = 2 cm}

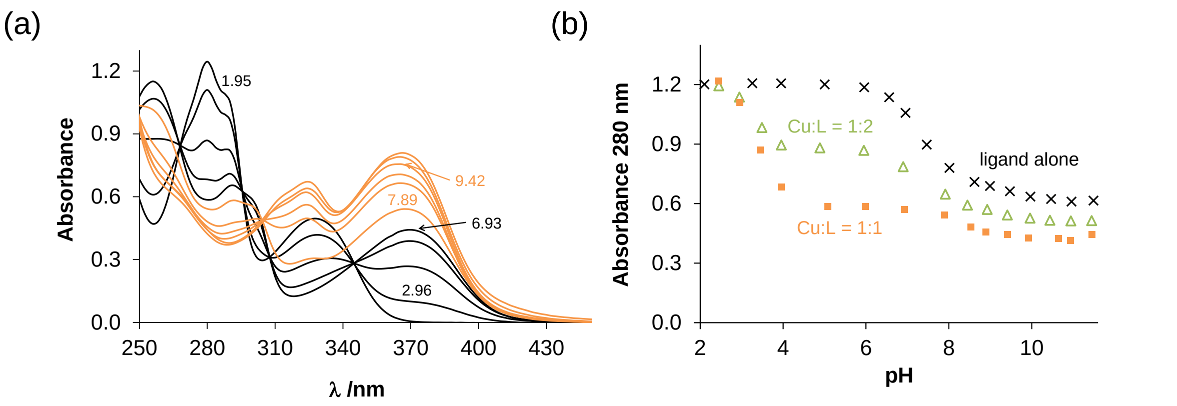

Figure S9. (**a**) UV-vis absorption spectra of the Cu(II) – Pro-SISC-Me (1:1) system recorded in water at various pH values, and (**b**) changes of the absorbance at 280 nm at 1:1 and 1:2 Cu(II)-to-ligand ratios and for the ligand alone. {c_{ligand} = 50 μM; T = 25 ˚C; I = 0.10 M (KCl); ℓ = 1 cm}

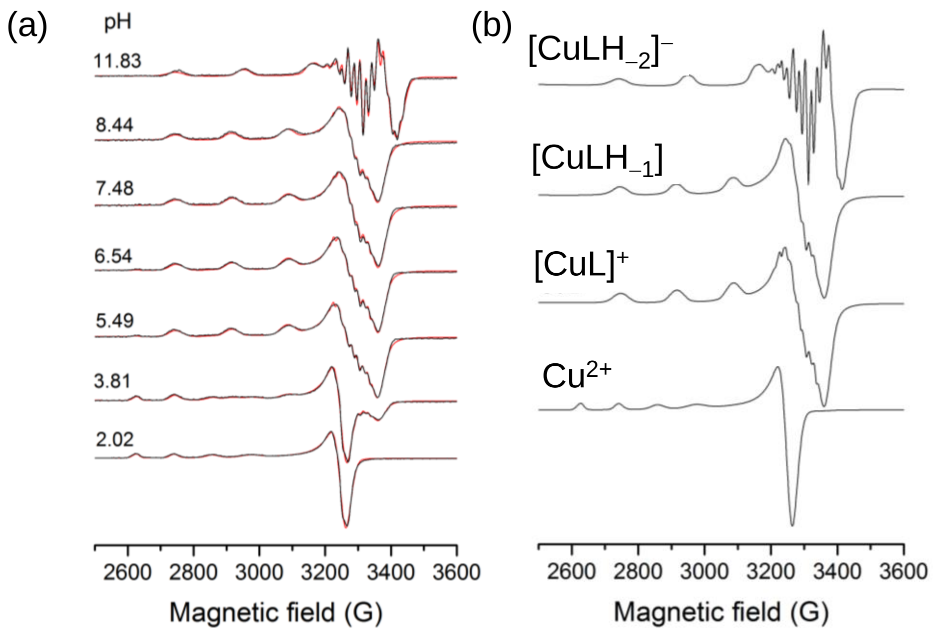

Figure S10. (**a**) Experimental (black) and simulated (red) anisotropic EPR spectra recorded for the Cu(II) – SISC (1 : 1) system in 30% (*v*/*v*) DMSO/H_2O at 77 K at various pH values, and (**b**) the calculated component frozen EPR spectra. {c_{ligand} = $c_{Cu(II)}$ = 250 μM; *I* = 0.10 M (KCl)}.

Table S1. Isotropic EPR parameters calculated for the various species in the Cu(II) – Pro-SISC-Me complexes in water, and the overall stability constants of the complexes calculated from the deconvolution of the spectra. {*I* = 0.10 M (KCl)}[a]

	**Cu^{2+}**	**$[CuL]^{+}$**	**$[CuLH_{-1}]$**	**$[CuLH_{-2}]^{-}$**
g_0	2.192	2.119	2.106	2.100
A_0 / G	36.6	61.6	66.2	86.6
a^{N1} / G	–	16.9	17.2	15.5
a^{N2} / G	–	12.1	12.2	14.1
α / G	48	25	25	23.4
β / G	–1	–13.3	–12.7	2.8
γ / G	1.5	2.7	2.8	3.0
**logβ**	–	14.78	6.50	–4.32

[a] The experimental errors were ±0.001 for g0 and ±1 G for couplings and linewith parameters.

Table S2. Anisotropic EPR parameters calculated for the various species in the Cu(II) – SISC complexes. {30% (*v*/*v*) DMSO/H_2O; *I* = 0.10 M (KCl)}[a]

	Cu^{2+}	$[CuL]^+$	$[CuLH_{-1}]$	$[CuLH_{-2}]^-$
g_x	2.080	2.039	2.045	2.033
g_y	2.080	2.059	2.048	2.045
g_z	2.410	2.248	2.245	2.206
$g_{0, calc.}$[b]	2.190	2.115	2.113	2.095
A_x / G	8	20	14	24
A_y / G	8	22	22	33
A_z / G	113	166	168	205
a_x^{N1} / G	–	9	10	18
a_y^{N1} / G	–	14	14	16
a_z^{N1} / G	–	11	10	14
a_x^{N2} / G	–	16	15	14
a_y^{N2} / G	–	8	16	22
a_z^{N2} / G	–	15	11	6

[a] The experimental errors were ±0.002 for g_x and g_y, ±0.001 for g_z, ±2 G for A_x and A_y, and ±1 G for A_z and nitrogen couplings. [b] Isotropic values of the *g* tensor are calculated via equation: $g_0 = (g_x + g_y + g_z)/3$.

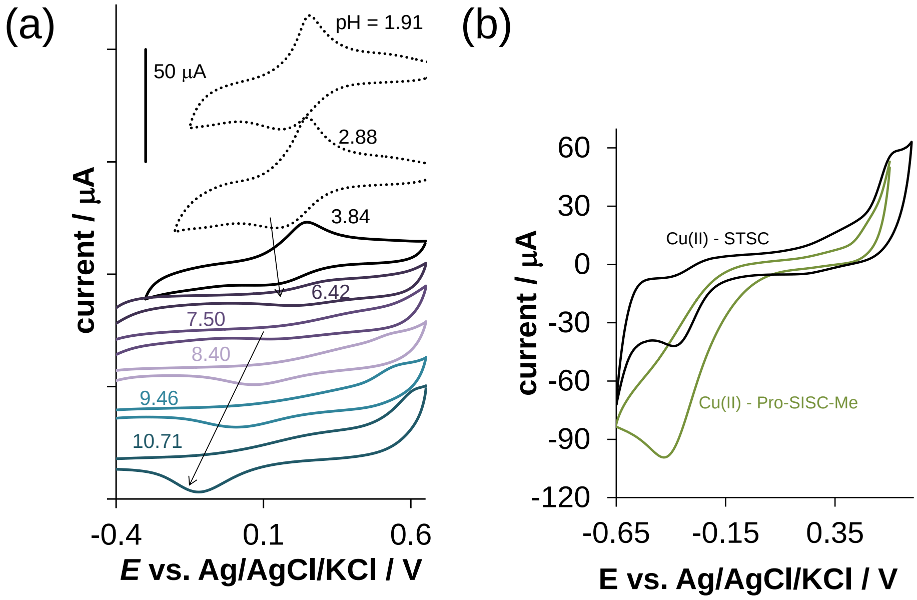

Figure S11. Cyclic voltammograms recorded for the (**a**) Cu(II) – SISC system at various pH values and for (**b**) Cu(II) – Pro-SISC-Me and Cu(II) – STSC systems at pH 7.4 at 1:1 metal-to-ligand ratio. {30% (*v*/*v*) DMSO/H_2O; $c_{ligand} = c_{Cu(II)}$ = 1 mM; *T* = 25 °C; *I* = 0.10 M (TBAN); scan rate: 20 mV/s}

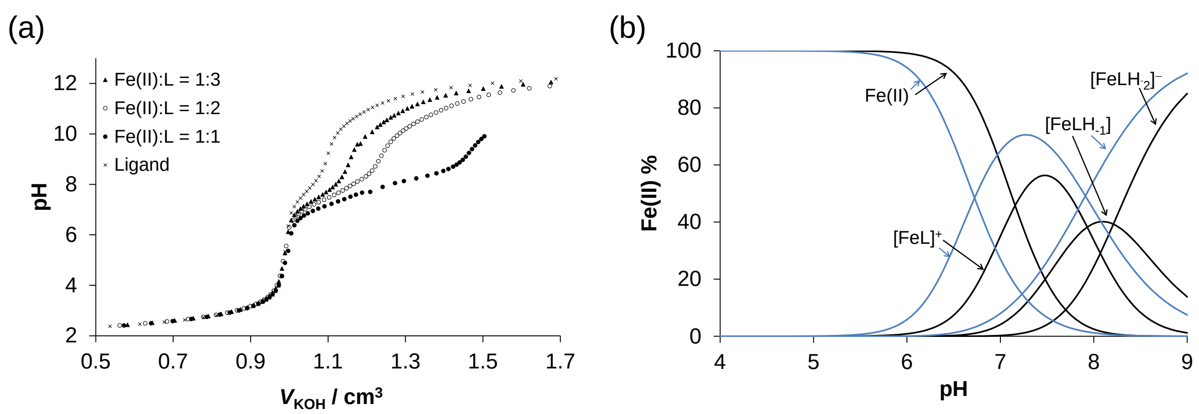

Figure S12. pH-potentiometric titration curves obtained for the (**a**) Fe(II) – SISC system in 30% (v/v) DMSO/H_2O at various metal-to-ligand ratios (indicated in the figure). (**b**) Concentration distribution curves for the Fe(II) – SISC (black lines) and Fe(II) – Pro-SISC-Me (blue lines) systems at 1:2 metal-to-ligand ratio. {c_{ligand} = 1 mM; T = 25 ˚C; I = 0.10 M (KCl)}

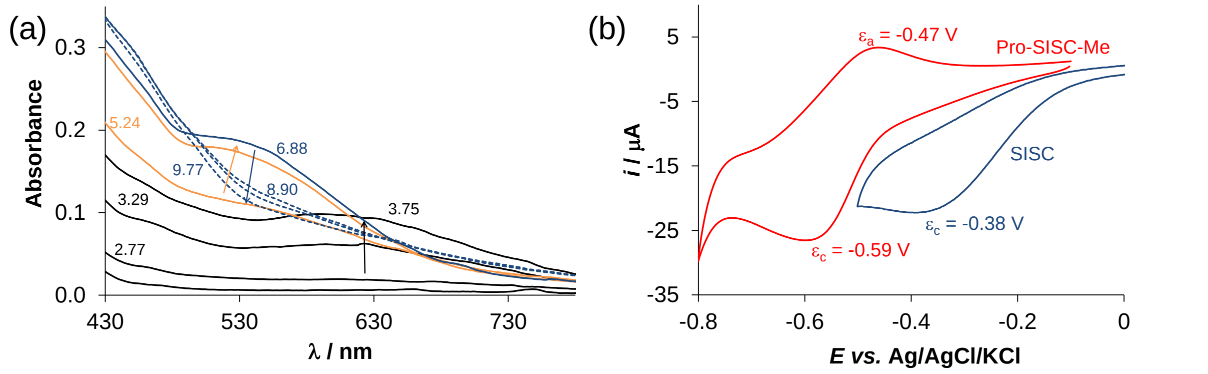

Figure S13. (**a**) UV-vis spectra recorded for the Fe(III) – SISC (1:2) system in 30% (v/v) DMSO/H_2O at various pH values (indicated in the figure). {c_{ligand} = 0.5 mM; T = 25 ˚C; I = 0.10 M (KCl); ℓ = 1 cm} (**b**) Cyclic voltammograms for the iron complexes of SISC (blue line) and Pro-SISC-Me (red line) at 1:2 metal-to-ligand ratio at pH 7.6 in 30% (v/v) DMSO/H_2O. {c_{ligand} = 1 mM; T = 25 ˚C; I = 0.10 M (TBAN)}

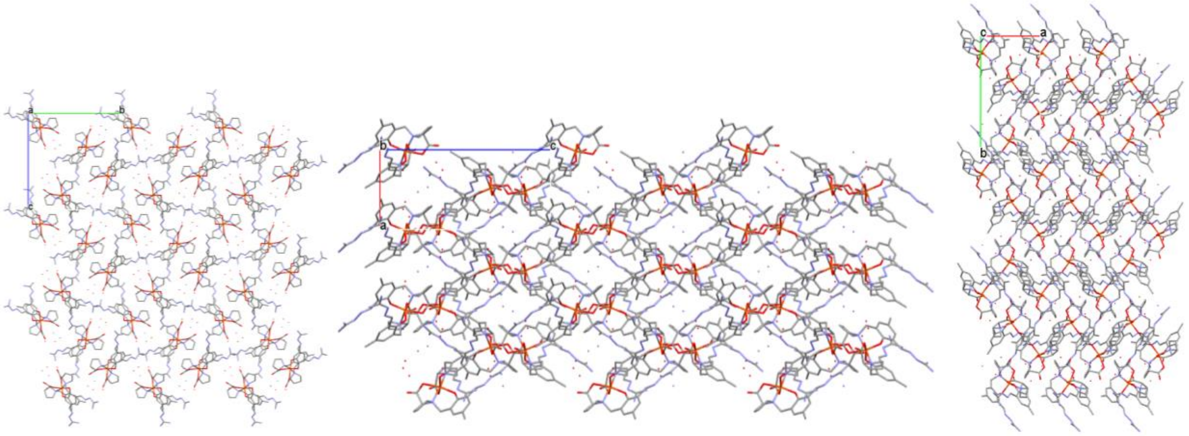

Figure S14. Packing arrangement of crystal (**2**) showing some selected hydrogen bonds and C-H…π interactions.

Table S3. Crystal data and details of data collection for Pro-SISC-Me·HBr·4H_2O (**1**)

Compound	**1**
Empirical formula	$C_{15}H_{30}BrN_5O_7$
Formula weight	472.35
Space group	orthorhombic, $P2_12_12_1$
Unit cell dimensions	
a (Å)	6.7067(2)
b (Å)	14.9720(4)
c (Å)	21.2674(5)
V (Å)	2135.52(10)
Z	4
λ (Å)	1.54178
Density (calculated) (mg/m^3)	1.469
Crystal size (mm)	0.28 × 0.03 × 0.02
Temperature (K)	100(2)
Absorption coefficient, μ (mm^{-1})	3.027
R_1[a]	0.0382
wR_2[b]	0.1003
Goodness-of-fit[c]	1.052

[a] $R_1 = \Sigma||F_o| - |F_c||/\Sigma|F_o|$. [b] $wR_2 = \{\Sigma[w(F_o^2 - F_c^2)^2]/\Sigma[w(F_o^2)^2]\}^{1/2}$. [c] Goodness-of-fit (GOF) = $\{\Sigma[w(F_o^2 - F_c^2)^2]/(n - p)\}^{1/2}$, where n is the number of reflections and p is the total number of parameters refined.

Table S4. Crystal data and structure refinement of Na[Fe(III)(Pro-SISC-MeH$_{-2}$)$_2$](H_2O)$_4$ (**2**)

Compound	**2**
Colour/shape	Red/Chunk
Empirical formula	$C_{30}H_{46}N_{10}O_{10}FeNa$
Moiety formula	[Fe($C_{30}H_{38}N_{10}O_6$)], 4(H_2O), Na
Formula weight	785.61
Radiation and wavelength (Å)	Mo-Kα, λ =0.71073
Crystal system	orthorhombic
Space group	$P2_12_12_1$
Unit cell dimensions	
a (Å)	9.361(2)
b (Å)	18.993(4)
c (Å)	20.041(4)
Volume (Å$^{3)}$	3562.9(14)
Z/*Z′*	4/1
Density (calculated) (mg/m^3)	1.465
Absorption coefficient, μ (mm^{-1})	0.505
Crystal size (mm)	0.60 x 0.10 x 0.05
Temperature (K)	103(2)
Absorption correction	Numerical
Data / restraints / parameters	6003 /12 /495
R_1[a]	0.1051
wR_2[b]	0.2268
Goodness-of-fit on *F*2 [c]	1.092

[a] $R_1 = \Sigma||F_o| - |F_c||/\Sigma|F_o|$. [b] $wR_2 = \{\Sigma[w(F_o^2 - F_c^2)^2]/\Sigma[w(F_o^2)^2]\}^{1/2}$. [c] Goodness-of-fit (GOF) = $\{\Sigma[w(F_o^2 - F_c^2)^2]/(n - p)\}^{1/2}$, where n is the number of reflections and p is the total number of parameters refined.
